# UGCG overexpression leads to increased glycolysis and increased oxidative phosphorylation of breast cancer cells

**DOI:** 10.1038/s41598-020-65182-y

**Published:** 2020-05-18

**Authors:** Nina Schömel, Lisa Gruber, Stephanie J. Alexopoulos, Sandra Trautmann, Ellen M. Olzomer, Frances L. Byrne, Kyle L. Hoehn, Robert Gurke, Dominique Thomas, Nerea Ferreirós, Gerd Geisslinger, Marthe-Susanna Wegner

**Affiliations:** 1 Pharmazentrum frankfurt/ZAFES, Institute of Clinical Pharmacology, Johann Wolfgang Goethe University, Theodor Stern-Kai 7, 60590 Frankfurt am Main, Germany; 2 School of Biotechnology and Biomolecular Sciences, University of New South Wales, Sydney, New South Wales 2052 Australia; 3 Fraunhofer Institute for Molecular Biology and Applied Ecology IME, Project Group Translational Medicine and Pharmacology (TMP), Theodor Stern-Kai 7, 60590 Frankfurt am Main, Germany

**Keywords:** Breast cancer, Glycobiology, Cancer metabolism

## Abstract

The only enzyme in the *glycosphingolipid* (GSL) metabolic pathway, which produces *glucosylceramide* (GlcCer) *de novo* is *UDP-glucose ceramide glucosyltransferase* (UGCG). UGCG is linked to pro-cancerous processes such as multidrug resistance development and increased proliferation in several cancer types. Previously, we showed an UGCG-dependent glutamine metabolism adaption to nutrient-poor environment of breast cancer cells. This adaption includes reinforced oxidative stress response and fueling the *tricarboxylic acid* (TCA) cycle by increased glutamine oxidation. In the current study, we investigated glycolytic and oxidative metabolic phenotypes following UGCG *overexpression* (OE). UGCG overexpressing MCF-7 cells underwent a metabolic shift from quiescent/aerobic to energetic metabolism by increasing both glycolysis and oxidative glucose metabolism. The energetic metabolic phenotype was not associated with increased mitochondrial mass, however, markers of mitochondrial turnover were increased. UGCG OE altered sphingolipid composition of the *endoplasmic reticulum* (ER)/mitochondria fractions that may contribute to increased mitochondrial turnover and increased cell metabolism. Our data indicate that GSL are closely connected to cell energy metabolism and this finding might contribute to development of novel therapeutic strategies for cancer treatment.

## Introduction

An essential hallmark of neoplastic diseases is persistent and uncontrolled cell proliferation. According adjustments of cellular energy metabolism provide, amongst others, sufficient macromolecule levels and energy in the form of *adenosine triphosphate* (ATP) to fuel cell growth and division. To understand the underlying molecular mechanisms of these metabolic changes is the first step to develop new therapeutic strategies for cancerous diseases. *Glycosphingolipids* (GSL) are not only important membrane components, but also act as signaling molecules in physiological and pathophysiological processes such as apoptosis and proliferation (reviewed in^1,2^). Numerous studies show specific expression of various GLS in particular cancers (reviewed in^2^) such as ganglioside GD2 in breast cancer^3^. Glycosylated sphingolipids cluster in the plasma membrane leading to the formation of *GLS enriched microdomains* (GEMs). These dynamic aggregations of sphingolipids, cholesterol and proteins are functional clusters and provide signaling platforms for membrane proteins, which are regulated by the lipid composition of the GEM (reviewed in^4^). Lipid microdomains are also present in the membranes of subcellular organelles modulating cytoplasmic pathways such as apoptosis (reviewed in^5^). Previous studies revealed that *UDP-glucose ceramide glucosyltransferase* (UGCG) *overexpression* (OE) leads to alterations of GEM composition in breast cancer cells resulting in signaling pathway activation and subsequently altered gene expression^6^. UGCG is a Golgi apparatus-residing enzyme that transfers an UDP-glucose molecule to ceramide to produce *glucosylceramide* (GlcCer), which is the precursor for all complex GSL. UGCG OE was reported in various cancers^7^ and is related to poor prognosis for breast cancer patients^8^ (reviewed in^9^). Otto Warburg was the first, who described aberrant characteristics of cancer cell energy metabolism as compared to non-tumor cells^10,11^. Specifically reprogramming of glucose metabolism to increased glycolysis, despite sufficient oxygen supply, and subsequent increased glucose consumption were observed in tumor tissues (reviewed in^12^). In the last years the attention was also drawn to mitochondria. Impairment of mitochondrial respiration was thought to be the reason for increased aerobic respiration of cancer cells and cancer development, but several studies showed that this is not the case for all cancer types (reviewed in^13^). Furthermore, it is now established that mitochondrial respiration defects are not generally the cause of reinforced aerobic glycolysis. Rather specific tumors, which are mostly glycolytic, retain a high mitochondrial respiration capacity (reviewed in^13^). Mitochondria are not only biosynthetic centers, for example by producing energy in form of ATP, but also are crucial signaling hubs. The organelles use various substrates from the cytoplasm to drive for example the *tricarboxylic acid* (TCA) cycle, mitochondrial membrane potential, fatty acid oxidation as well as *de novo* lipid synthesis (reviewed in^13^). *Reactive oxygen species* (ROS), which are mostly generated as a biproduct of the electron transport chain, are pro-tumorigenic and elevated levels are associated with cancer (reviewed in^14^). But ROS also act as signaling molecules for example by *hypoxia inducible factor-1* (HIF-1) activation, which influences cellular proliferation^15^. Furthermore, mitochondria are important apoptosis regulators via the *B-cell lymphoma protein* (Bcl-2) family and associated proteins^16^ and maintain calcium homeostasis^17^. While most mitochondrial proteins are encoded by nuclear genes, mitochondria possess a small DNA genome (mtDNA) that encodes proteins essential for respiration, transfer RNAs and ribosomal RNAs. Mitochondrial morphology is regulated by various cellular pathways like *mitogen activated protein kinases* (MAPK), *phosphoinositid-3-Kinase*-Akt (PI_3_K-Akt) and *myelocytomatosis* (MYC) (reviewed in^18^). They form a network of long interconnected tubules and continually undergo fission and fusion. Mitochondria share nutrients, mtDNA and electron transport chain components by fusion and they divide to be distributed to daughter cells during mitosis or to be able to migrate to regions of higher energy demand (reviewed in^18^). Fission additionally facilitates mitophagy (reviewed in^18^). Mitochondria are tightly associated with membrane structures of the *endoplasmic reticulum* (ER). It was shown that these contact sites are functionally linked to diverse physiologic processes such as ATP production, apoptosis and mitochondrial dynamics (reviewed in^5^). Several studies have proven that alterations of mitochondrial biogenesis, dynamics and degradation are linked to diverse pathologies including cancer progression. Novel diagnostic and therapeutic approaches are already targeting mitochondrial redox homeostasis, TCA cycle, *oxidative phosphorylation* (OXPHOS) proteins or mitochondrial dynamics (reviewed in^13^). One example is *dynamin related protein1* (DRP1), whose inhibition is currently under investigation. DRP1 is essential for mitochondrial fission and its blocking leads to reduced growth of glioblastoma cancer stem cells^19^ *in vitro* and lung adenocarcinoma cells *in vivo*^20^. In addition, DRP1 blockage inhibits tumor sphere formation of breast cancer and melanoma cells^21^. Since we discovered that OE of UGCG, a key enzyme of GSL metabolism, leads to increased cellular proliferation of breast cancer cells^6^, we were interested in the molecular mechanisms which link GSL to cellular energy metabolism. In previous studies, we were able to show that glutamine is used for reinforced oxidative stress response via glutathione production and fuels the TCA cycle to sustain the proliferative advantage of UGCG overexpressing breast cancer cells^22^.

In the current study we show that UGCG OE increases substrate oxidation and OXPHOS in breast cancer cells. OE of UGCG transforms cells from a quiescent/aerobic state to an energetic state, which is not ascribable to increased mitochondrial mass. Alterations of the sphingolipid composition of the ER/mitochondria fractions seem to influence OXPHOS protein activity leading to increased OXPHOS, which is accompanied by an accelerated mitochondrial turnover. Our data indicate that GSL are closely connected to cell energy metabolism, which is to our knowledge a novel finding. This finding might contribute to development of novel therapeutic strategies for cancer treatment.

## Material and Methods

### Cell culture

Human MCF-7 breast cancer cell line was obtained from the Health Protection Agency (European Collection of Cell Cultures EACC, Salisbury, UK). Cells were cultured as described previously^22^ and stably transfected cells were selected by supplementing 200 µg/ml G418 (Thermo Fisher Scientific, Waltham, USA).

### Generation of stable UGCG expressing cells

The UGCG expression plasmid (pCMV6-ENTRY vector, OriGene Technologies Inc., Rockville, USA) (MCF-7/UGCG OE) and the empty control plasmid (MCF-7/empty) were stably transfected into MCF-7 cells using Lipofectamine 2000 (Invitrogen, Carlsbad, USA), transfected cells were selected over five weeks using G418 as previously described^6^.

### Substrate competition assay

Tracer measurements were performed as described in^23^. Briefly, 1.5 ×10^4^ cells/24 well were seeded in cell culture media (a day prior to the assay) and then incubated in *Krebs Ringer Phosphate* (KRP) nutrient buffer containing either D-[3-^3^H] glucose or D-[^14^C (U)] glucose. Substrate oxidation was measured capturing evolved ^14^CO_2_. For glycolysis measurements, D-[3-^3^H] glucose was separated from tritiated [^3^H]_2_O by diffusion. To quantify tracer oxidation, media was acidified and ^14^CO_2_ trapped via reaction with 0.1 ml KOH before liquid scintillation spectrometry. To quantify tracer incorporation into cellular lipids, a chloroform–methanol (2:1 vol./vol.) extraction was performed and fractions assayed by scintillation spectrometry.

### Measurement of mitochondrial respiration and glycolysis

The Seahorse XFe Analyzer (Agilent Technologies, Santa Clara, USA) was used to simultaneously measure the *oxygen consumption rate* (OCR) and the *extracellular acidification rate* (ECAR) in real-time. Cells were seeded and treated as described in^24^. Instead of *carbonyl cyanide m-chlorophenylhydrazone* (CCCP), cells were treated with 10 µM *N*^5^*,N*^6^*-*bis*(2-fluorophenyl)-[1,2,5]oxadiazolo[3,4-b]pyrazine-*5,6*-diamine* (BAM15) (Cayman Chemical, Ann Arbor, USA).

### Quantification of mitochondrial ROS

0.5 ×10^4^ cells/96-well (black) were seeded and incubated for 24 h at 37 °C. Subsequently, cells were incubated with 5 μM Red Mitochondrial Superoxide Indicator for live-cell imaging (MitoSOX) (Thermo Fisher Scientific, Waltham, USA) for 30 min at 37 °C. Following a three-time washing step with PBS, fluorescence was detected on a Fluostar plate reader with excitation at 510 nm and emission at 590 nm. The values were background corrected (only medium).

### Comparison of mitochondrial mass using nonyl acridine orange (NAO)

Cells were seeded in 6 cm dishes and treated with 100 nM NAO the following day. After 15 minutes of incubation, cells were harvested by trypsin and pelletized. After one washing step in in PBS, mitochondrial staining of 100,000 cells per sample was analyzed via FITC channel with the BD FACS Canto II flow cytometer and the BD FACSDiva software (BD Biosciences, Franklin Lakes, USA). Data were evaluated using FlowJo software (FlowJo LLC, Ashland, USA) and related to MCF-7/empty control cells.

### Protein concentration determination by Western blot analysis

Western Blot analysis was applied to investigate the relative levels of the five mitochondrial OXPHOS complexes and mitochondrial fission and fusion enzymes of MCF-7/empty and UGCG overexpressing cells. Cells were harvested and the pellet was resuspended in PhosphoSafe buffer (EMD Chemicals Inc. Billerica, USA), 2 mM DTT (AppliChem GmbH, Darmstadt, Germany), 1 x Roche Complete (Roche, Mannheim, Germany), pH 7.4 supplemented with 1% 100 X Halt Protease Inhibitor Cocktail (Thermo Fisher Scientific, Darmstadt, Germany). After sonification, the lysate was centrifuged (14,000 x g, 10 min, 4 °C). The protein concentration in the supernatant was determined via the Bradford method. 40–75 µg total protein extract (heated only at 50 °C for 5 minutes because of the heat sensitivity of the Complex IV unit) were separated by 12 or 15% SDS-PAGE and electro-blotted onto a nitrocellulose membrane (Amersham Protran, GE Healthcare Life Sciences, Freiburg, Germany). Ponceau staining (0.5% in 1% acetic acid) was applied to verify protein transfer. After 90 minutes incubation in 5% milk powder diluted in *PBS with 0.1% Tween* 20 (PBST) for OXPHOS proteins and 1:1 dilution of PBST and Intercept (PBS) Blocking Buffer for fission and fusion enzymes (LI-COR Biosciences, Bad Homburg, Germany), the membrane was incubated with the primary antibody (Total OXPHOS Rodent WB Antibody Cocktail (ab110413, Abcam, Cambridge, UK) (1:250 in 1% milk powder in PBST, two days, 4 °C) or antibodies from the Mitochondrial Dynamics Antibody Sampler Kit (#48799 Cell Signaling Technology, Cambridge, UK) (1:1000 (MFN1, MFN2, OPA1, MFF, DRP1 and TOM20) or 1:200 (Phospho-DRP1 (Ser616)) in 1:1 dilution of PBST and Intercept (PBS) Blocking Buffer (LI-COR Biosciences, Bad Homburg, Germany). The IRDye680 conjugated secondary antibody (LI-COR Biosciences, Bad Homburg, Germany), was used for all proteins except phospho-DRP1 (Ser616) (1:10,000 in blocking solution, one hour at RT). Fluorescence emission and densitometric analysis was performed using the Odyssey Infrared Scanner (LI-COR Biosciences, Bad Homburg, Germany) and the Image Studio Lite software (LI-COR Biosciences, Bad Homburg, Germany). The protein concentration was related to Ponceau staining intensity or *heat shock protein 90* (HSP90) as a housekeeping protein (1:1000; 30 minutes incubation (BD Biosciences, Franklin Lakes, New Jersey, USA), secondary antibody IRDye 800 conjugated (LI-COR Biosciences, Bad Homburg, Germany), 60 minutes incubation). For phospho-DRP1 (Ser616) protein detection, the anti-rabbit IgG, HRP-linked secondary antibody (#7074 Cell Signaling Technology, Cambridge, UK) and the Anti-Mouse IgG (whole molecule)–Peroxidase antibody (produced in rabbit, #A9044, Sigma-Aldrich, St. Louis, USA) for HSP90 detection was applied. Protein concentration was detected via enhanced chemiluminescence method using Pierce ECL Western Blotting Substrate (#32106, Thermo Fisher Scientific, Waltham, USA).

### Analysis of mitochondrial DNA copy numbers per cell

The NovaQUANT Human Mitochondrial to Nuclear DNA Ratio Kit (Merck KGaA, Darmstadt, Germany) and SYBR select Master Mix (Thermo Fisher Scientific, Waltham, USA) were used to compare the nuclear to mitochondrial DNA ratio. The qRT-PCR based kit contains primer pairs targeting two mitochondrial (ND1 and ND6) and two nuclear genes (BECN1 and NEB). By calculating ratios of the Ct values of BECN1/ND1 and NEB/ND6, the mtDNA copy number per cell was determined. DNA was isolated using KAPA Express Extract Kit (Kapa Biosystems, Wilmington, USA) and 2 ng DNA per reaction well were applied.

### Quantitative real-time PCR (qRT-PCR)

Quantitative real-time PCR (qRT-PCR) was performed as described previously^22^. Briefly, the RNeasy Mini Kit (QIAGEN, Hilden, Germany) was used to isolate total RNA. 300 ng RNA were applied to synthesize cDNA using the Verso cDNA Synthesis Kit (Thermo Fisher Scientific, Waltham, USA). Gene-specific PCR products were quantified utilizing 5 X QPCR Mix EvaGreen (ROX) (Bio&SELL, Feucht, Germany) on a QuantStudio 5 Real-time PCR System (Thermo Fisher Scientific, Waltham, USA). Relative mRNA expression was calculated according to the Δct method. Relative values were normalized to *60 S ribosomal protein L37a* (RPL37A) expression level housekeeping gene. All primers, as listed in Table 1, were purchased from Eurofins (Luxembourg, Luxembourg).

**Table 1:** Primer for qRT-PCR.

Gene	Forward primer 5′ → 3′	Reverse primer 5′ → 3′	Amplicon (bp)
RPL37A	attgaaatcagccagcacgc	aggaaccacagtgccagatcc	94
MFN1	atgacctggtgttagtagacagt	agacatcagcatctaggcaaaac	90
MFN2	cacatggagcgttgtaccag	ttgagcacctccttagcagac	104
PARK2	gtgtttgtcaggttcaactcca	gaaaatcacacgcaactggtc	129
FIS1	gatgacatccgtaaaggcatcg	agaagacgtaatcccgctgtt	82
PINK	cccaagcaactagcccctc	ggcagcacatcagggtagtc	107
OGDH	tgccagcatattggggtgg	ggaactcctcaaacctggtgg	160
PDHA1	tggtagcatcccgtaattttgc	attcggcgtacagtctgcatc	151
PDHB	aagaggcgctttcactggac	actaaccttgtatgccccatca	153
GOT1	atttcttagcgcgttggtaca	acacagcattgtgattctccc	90
GOT2	aagagtggccggtttgtcac	agaaagacatctcggctgaact	107
LDHA	atggcaactctaaaggatcagc	ccaaccccaacaactgtaatct	86
GOLPH3	aggaagccgttcttgacaaatg	ggcatgagccaggtaaatgag	83
OPA1	tcaagaaaaacttgatgctttca	gcagagctgattatgagtacgatt	78

### Measurement of mitochondrial membrane potential

To determine mitochondrial membrane depolarization of MCF-7/UGCG OE and MCF-7/empty cells, the cationic dye JC-1 that exhibits potential-dependent accumulation in mitochondria, was applied. Cells were seeded in 6 cm dishes and the next day, at 80% confluency, cells were stimulated with 2 µg/ml JC-1 for 20 minutes. Subsequently, cells were washed with PBS, harvested by trypsin and pelletized. The pellet was resuspended in 250 µl PBS and 100,000 cells per sample were measured using the BD FACS Canto II flow cytometer (monomer form: 514/529 nm; J-aggregate form: 585/590 nm) and the BD FACSDiva software (BD Biosciences, Franklin Lakes, USA). Data were evaluated using FlowJo software (FlowJo LLC, Ashland, USA).

### Sphingolipid level analysis of ER/mitochondria fractions

To analyze the lipid composition of the ER/mitochondria fractions, mitochondria from 1.5 ×10^7^ freshly harvested cells were isolated using the Qproteome Mitochondria Isolation Kit (Cat. no. 37612, QIAGEN, Hilden, Germany). All steps were conducted according to the manufacturer’s protocol not including the high-purity preparations. The fraction purity was confirmed via Western Blot analysis. The sphingolipid concentrations of the isolated ER/mitochondria fractions were determined by *liquid chromatography tandem mass spectrometry* (LC-MS/MS) as described previously^6^. Levels are related to the protein concentrations of the cytosolic fractions and normalized to MCF-7/empty control cells.

### Statistical analysis

Statistical analysis was performed with GraphPad Prism 7 software. Data are presented as mean ± *standard error of the mean* (SEM). Significant differences in means between two groups were assessed by unpaired *t-*test with Welch’s correction and by two-way ANOVA with a Bonferroni multi-comparison post-test for more than two groups.

### Ethical approval

This article does not contain any studies with human participants or animals performed by any of the authors.

## Results

### UGCG overexpression leads to a metabolic shift from an aerobic/quiescent to an energetic cell type

**Figure 1:**
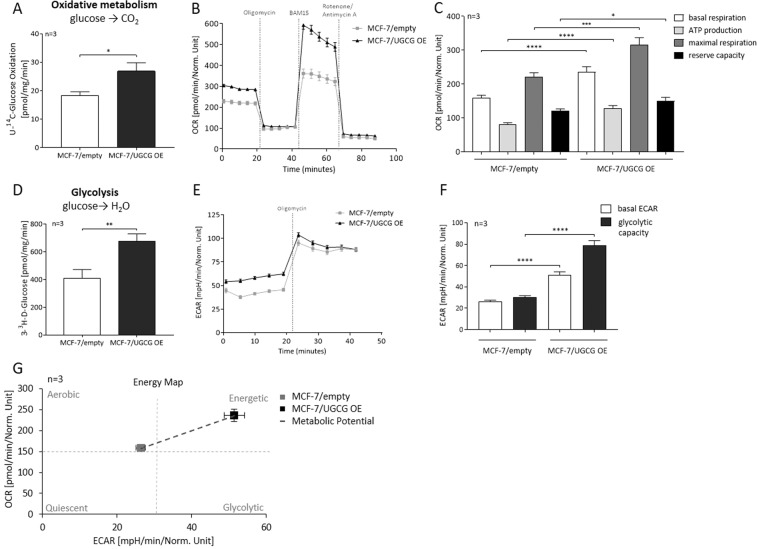
UGCG overexpression enhances glycolysis and cellular respiration. (**A**) Substrate oxidation was measured by U-^14^C-glucose tracing experiments. The evolved ^14^CO_2_ was determined. Data are presented as mean of n = 3 ± *standard error of the mean* (SEM). (**B**) Representative graph of the *oxygen consumption rate* (OCR) determined by Seahorse XFe analyzer. (**C**) ATP production (basal respiration − respiration after oligomycin injection), maximal respiration rate (respiration after *N5,N6-bis(2-fluorophenyl)-[1,2,5]oxadiazolo[3,4-b]pyrazine-5,6-diamine* (BAM15) injection − respiration after antimycin a/rotenone injection), reserve capacity (respiration after BAM15 injection − basal respiration). Data are presented as mean of n = 3 ± SEM. (**D**) Anaerobic glycolysis was determined by using 3-^3^H-D-glucose and measuring the released labelled H_2_O. Data are presented as mean of n = 3 ± SEM. (**E**) Representative graph of the *extracellular acidification rate* (ECAR) quantified by Seahorse XFe analyzer. (**F**) Glycolytic capacity (deduced from ECAR after oligomycin treatment − basal ECAR). Data are presented as mean of n = 3 ± SEM. (**G**) Energy map of MCF-7/UGCG OE and control cells. *p ≤ 0.05, **p ≤ 0.01, ***p ≤ 0.001, ****p ≤ 0.0001.

In previous studies we showed increased glutamine uptake and reinforced glutamine oxidation following UGCG *overexpression* (OE) in MCF-7 cells^22^. In this study we performed a substrate competition assay with [U-^14^C]-labelled glucose to track the metabolism of glucose-derived carbons in MCF-7/UGCG OE and control cells. UGCG OE led to an increased level of captured ^14^CO_2_, which demonstrates that UGCG OE increases glucose oxidation (Fig. 1A). The basal mitochondrial respiration, which is represented by *oxygen consumption rate* (OCR), was significantly increased in MCF-7/UGCG OE cells as compared to control cells (Fig. 1B,C). Likewise, ATP production (basal respiration − respiration after oligomycin injection) was significantly increased and the maximal respiration rate (respiration after *N*^*5*^*,N*^*6*^*-*bis*(2-fluorophenyl)-[1,2,5]oxadiazolo[3,4-b]pyrazine-5,6-diamine* (BAM15) injection − respiration after antimycin a/rotenone injection) was significantly increased in MCF-7/UGCG OE cells as compared to control cells (Fig. 1C). The maximal respiration indicates the ability of the cellular respiration to respond to an increased energy demand. Additionally, the reserve capacity (respiration after BAM15 injection − basal respiration) was significantly increased in MCF-7/UGCG OE cells as compared to control cells (Fig. 1C). The addition of 3-^3^H-D-glucose to the media and subsequent measurement of evolved ^3^H_2_O shows increased glycolysis following UGCG OE (Fig. 1D). In line with these data, the basal *extracellular acidification rate* (ECAR) (determined by the net production and extrusion of protons into the medium during lactate production from glucose) was significantly increased in UGCG overexpressing cells (Fig. 1E,F). Moreover, the glycolytic capacity (deduced from ECAR after oligomycin treatment − basal ECAR) of MCF-7/UGCG OE cells was significantly increased as compared to control cells (Fig. 1F).

In summary, following UGCG OE, MCF-7 cells transform from an aerobic/quiescence state to an energetic metabolic state (shift of metabolic potential) showed by increased glycolysis and oxidative phosphorylation (Fig. 1G).

### UGCG overexpression leads to increased superoxide levels and does not impact mitochondrial mass

**Figure 2:**
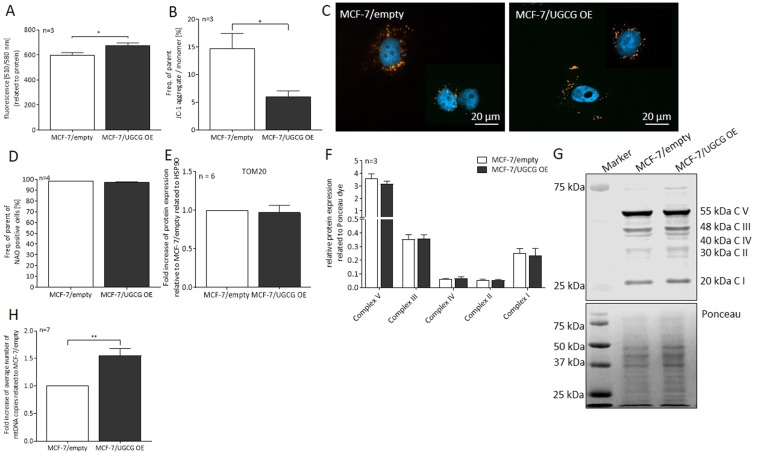
Influence of UGCG overexpression on mitochondrial ROS and mass. (**A**) Mitochondrial *reactive oxygen species* (ROS) levels were quantified using the fluorogenic reagent MitoSOX. Data are presented as a mean of n = 3 ± SEM. (**B**) Mitochondrial membrane potential was analyzed by flow cytometry utilizing *5,5,6,6’-tetrachloro-1,1’,3,3’ tetraethylbenzimidazoyl-carbocyanine iodide* (JC-1). Data are presented as a mean of n = 3 ± SEM. (**C**) Representative images of n = 3 of JC-1 staining by immunocytochemistry. From each of the biological samples at least 5 images were generated. (**D**) Mitochondrial mass determined by flow cytometry using *nonyl acridine orange* (NAO). Data are presented as a mean of n = 4 ± SEM. (**E**) Analysis of *translocase of the outer mitochondrial membrane 20* (TOM20) protein concentration by Western blot analysis. Protein expression is related to the housekeeper Hsp90 and control cells. Data are presented as a mean of n = 6 ± SEM. (**F**) Densitometrical analysis of *oxidative phosphorylation* (OXPHOS) complexes I-V protein concentrations by Western blot analysis. Protein expression is related to Ponceau dye. Data are presented as a mean of n = 3 ± SEM. (**G**) Representative image of the OXPHOS protein Western Blot analysis (upper part) and Ponceau staining (lower part). (**H**) The *mitochondrial DNA* (mtDNA) copy number was determined using the NovaQUANT Human Mitochondrial to Nuclear DNA Ratio Kit. Data are related to MCF-7/empty and presented as a mean of n = 7 ± SEM. *p ≤ 0.05, **p ≤ 0.01.

UGCG OE resulted in increased mitochondrial superoxide levels detectable as increased fluorescence of MitoSOX red dye (Fig. 2A). In addition, the mitochondrial membrane potential sensor *5,5,6,6’-tetrachloro-1,1’,3,3’ tetraethylbenzimidazoyl-carbocyanine iodide* (JC-1) was detected by flow cytometry. We measured a significant decrease of JC-1 aggregate to monomer ratio, which implies an increase in mitochondrial depolarization (Fig. 2B). A representative image of mitochondrial JC-1 staining is shown in Fig. 2C. To exclude the possibility that the metabolic effects are mitochondrial mass dependent, we analyzed mitochondrial mass by several parameters in MCF-7/UGCG OE and control cells. Since the MitoTracker green reacts with ROS, we used the fluorescent mitochondrial dye *nonyl acridine orange* (NAO) for mitochondrial mass analysis^25^. We labelled the cells with NAO and analyzed vital mitochondria by flow cytometry. No significant differences between UGCG overexpressing and control cells could be detected (Fig. 2D). The protein level of the receptor *translocase of the outer mitochondrial membrane* 20 (TOM20), which is a part of the TOM complex that enables the import of proteins through the outer mitochondrial membrane, was not changed upon UGCG OE (Fig. 2E and supplemental data 1). The OXPHOS protein content was not significantly altered in UGCG overexpressing cells as compared to control cells (Fig. 2F,G and supplemental data 2). The results are confirmed by UGCG knockdown (Kd) experiments. MCF-7/UGCG Kd cells exhibit no significant altered total OXPHOS protein content, whereas significantly reduced complex V protein (ATP synthase) concentration could be detected (supplemental data 3). On the contrary, we detected a significantly increased mtDNA copy number in MCF-7/UGCG OE cells in comparison to control cells (Fig. 2H).

Accordingly, our effects following UGCG OE are not ascribable to increased mitochondrial mass but might be due to increased OXPHOS protein activity.

### Mitochondrial turnover is accelerated following UGCG overexpression

**Figure 3:**
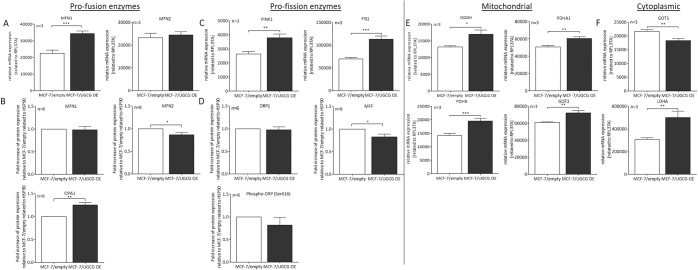
UGCG overexpression impact on mitochondrial dynamics and mitochondrial and cytoplasmic enzymes. (**A**) The mRNA expression of the pro-fission enzymes *mitofusin 1* (MFN1) and *mitofusin 2* (MFN2) was determined by qRT-PCR analysis. The expression is related to the housekeeping gene (RPL37A). Data are presented as a mean of n = 3 ± SEM. (**B**) Protein concentration of MFN1, MFN2 and *optic atrophy type 1* (OPA1) determined by Western blot analysis. Data are related to the housekeeping protein HSP90 and presented relative to MCF-7/empty control cells as a mean of n = 6 ± SEM. (**C**) mRNA levels of the pro-fission enzymes *PTEN-induced kinase 1* (PINK1) and *mitochondrial fission protein 1* (FIS1) related to RPL37A. Data are presented as a mean of n = 3 ± SEM. (**D**) Protein concentration determination of total *dynamin related protein1* (DRP1), total *mitochondrial fission factor* (MFF) and phospho-DRP1 (Ser616) by Western blot analysis. Data are related to the housekeeping protein HSP90 and presented relative to MCF-7/empty control cells as a mean of n = 6 ± SEM. (**E**) mRNA expression analysis of *alpha-ketoglutarate dehydrogenase* (OGDH), *pyruvate dehydrogenase E1 component subunit alpha* (PDHA1), and *beta* (PDHB) and *glutamate oxaloacetate transaminase 2* (GOT2) by qRT-PCR. The expression is related to RPL37A. Data are presented as a mean of n = 3 ± SEM. (**F**) mRNA expression analysis of *glutamate oxaloacetate transaminase 1* (GOT1) and *lactate dehydrogenase subunit A* (LDHA) by qRT-PCR. The expression is related to RPL37A. Data are presented as a mean of n = 3 ± SEM. *p ≤ 0.05, **p ≤ 0.01, ***p ≤ 0.001.

Next, we analyzed the expression of enzymes, which are involved in the process of mitochondrial fusion and fission by qRT-PCR and Western Blot analysis. We detected a significant increase of the mRNA level of *mitofusin 1* (MFN1) in MCF-7/UGCG OE cells, whereas *mitofusin 2* (MFN2) mRNA expression was unchanged (Fig. 3A). Contradictory, MFN1 protein level was not changed following UGCG OE (Fig. 3B and supplemental data 4), whereas MFN2 protein content was significantly decreased (Fig. 3B and supplemental data 5). Protein concentration of *optic atrophy type 1* (OPA1) was significantly increased in MCF-7/UGCG OE cells as compared to control cells (Fig. 3B and supplemental data 6) and the results are confirmed on mRNA level (supplemental data 7). The mRNA level of the pro-fission enzymes *PTEN-induced kinase 1* (PINK1), which accumulates in dysfunctional mitochondria, and *mitochondrial fission protein 1* (FIS1) were significantly increased following UGCG OE (Fig. 3C), whereas no significant difference on *Parkin 2* (PARK2) mRNA level could be detected (supplemental data 7). In addition, we determined total *dynamin related protein 1* (DRP1) and total *mitochondrial fission factor* (MFF) and *phospho-DRP1* (Ser616) protein level by Western blot analysis. No difference on total DRP1 (supplemental data 8) and phopho-DRP1 (Ser616) (supplemental data 9) protein level following UGCG OE could be detected (Fig. 3D). Total MFF protein levels are significantly reduced following UGCG OE (Fig. 3D and supplemental data 10). Phospho-MFF (Ser146) could not be detected (data not shown). Following Kd of UGCG in MCF-7 cells no significant differences on MFN1, PINK1, FIS1 and OPA1 mRNA expression levels are detectable as compared to control cells (supplemental data 11), which confirms UGCG-dependent increased mRNA expression of several proteins involved in the process of mitochondrial fusion and fission. In previous studies, UGCG OE led to an increase in glutamine metabolism in the TCA cycle^22^. To verify this data, we determined the mRNA levels of several mitochondrial enzymes involved in pyruvate processing and TCA cycle. The expression of the mitochondrial redox sensor *alpha-ketoglutarate dehydrogenase* (OGDH) is significantly increased in MCF-7/UGCG OE cells compared to MCF-7/empty control cells (Fig. 3E). OGDH catalyzes the conversion of alpha-ketoglutarate into succinyl CoA, which is the first step of the TCA cycle starting from glutamine. The *pyruvate dehydrogenase E1 component subunit alpha* (PDHA1) and *-beta* (PDHB) are units of the pyruvate dehydrogenase complex, which converts pyruvate to acetyl-CoA in the mitochondrial matrix. The mRNA level of both subunits is significantly increased in MCF-7/UGCG OE cells compared to MCF-7/empty control cells (Fig. 3E). The mitochondrial isoform 2 of the *glutamate oxaloacetate transaminase* (GOT2) is also significantly increased following UGCG OE (Fig. 3E) matching the increased aspartate metabolization from glutamate, which was shown by ^13^C_5_-glutamine labelling assay^22^. There is a significant decrease of the cytosolic *GOT isoform 1* (GOT1) mRNA level in MCF-7/UGCG OE cells compared to MCF-7/empty cells (Fig. 3F). Furthermore, a significant increase of the cytosolic *lactate dehydrogenase subunit A* (LDHA) mRNA level following UGCG OE was measured (Fig. 3F). LDHA catalyzes the conversion of pyruvate to lactate. The increase is in line with the elevated ECAR in MCF-7/UGCG OE cells (Fig. 1E,F).

### UGCG leads to alterations of sphingolipid composition in the ER/mitochondria fraction

**Figure 4:**
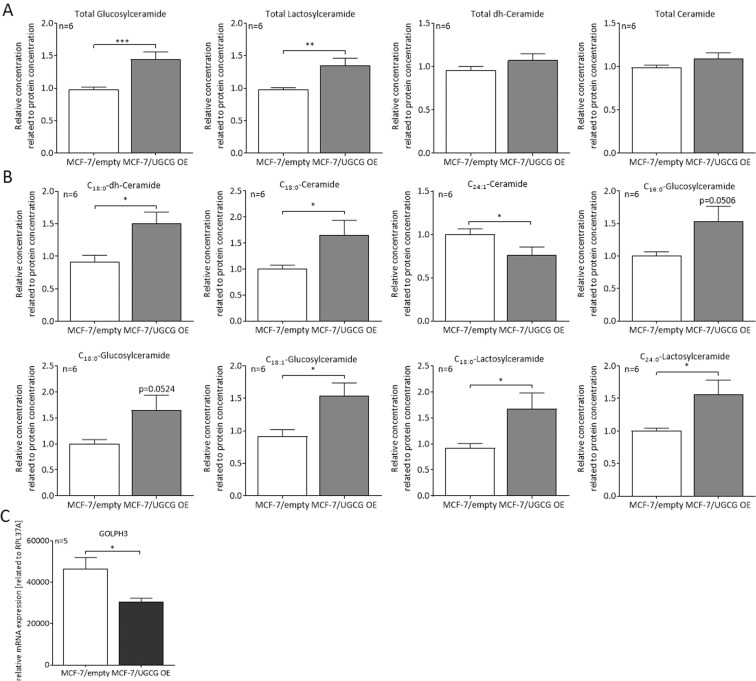
UGCG influences the lipid composition of ER/mitochondria fractions. (**A**) Total glucosylceramide, lactosylceramide, *dihydro-* (dh-) ceramide and ceramide levels of isolated ER/mitochondria fractions determined via LC-MS/MS. Data are presented as a mean of n = 6 ± SEM. (**B**) Levels of specific sphingolipids species following UGCG OE. Data are presented as a mean of n = 6 ± SEM. (**C**) *Golgi phosphoprotein 3* (GOLPH3) mRNA level related to RPL37A determined by qRT-PCR. Data are presented as a mean of n = 5 ± SEM. *p ≤ 0.05, ***p ≤ 0.001.

To explain the observed changes in mitochondria of MCF-7/UGCG OE cells, we compared the sphingolipid composition in *endoplasmic reticulum* (ER)/mitochondria fractions of MCF-7/UGCG OE and control cells. Since the ER is the place of *de novo* ceramide synthesis and ER and mitochondria are connected via specific ER-mitochondria contact sites, we did not isolate single ER and mitochondria fractions. Rather, we isolated ER/mitochondria fraction and the purity of the fraction was analyzed by Western blot analysis. The Western blot analysis results show high purity by *protein disulfate-isomerase* (PDI) (ER marker) and OXPHOS protein detection (supplemental data 12). Using *liquid chromatography tandem mass spectrometry (*LC-MS/MS), the sphingolipid levels of the ER/mitochondria fractions were quantified. A significant increase of total GlcCer (1.44-fold) and total *lactosylceramide* (LacCer) levels (1.35-fold) in MCF-7/UGCG OE cells as compared to control cells could be detected (Fig. 4A). The total *dihydro* (dh)-ceramide and total ceramide levels are unchanged following UGCG OE (Fig. 4A). In detail, the C_18:0_-dh-Cer, C_18:0_-Cer, C_16:0_-, C_18:0_-, C_18:1_-GlcCer and C_24:0_-LacCer levels are significantly increased in MCF-7/UGCG OE cells as compared to control cells (Fig. 4B). The C_18:0_-LacCer level is increased by tendency (p = 0.0512) in MCF-7/UGCG OE cells as compared to control cells (Fig. 4B). C_24:1_-Cer is the only sphingolipid, which is decreased in MCF-7/UGCG OE as compared to control cells (0.76-fold). All other analyzed sphingolipid concentrations are not significantly changed following UGCG upregulation (supplemental data 13). Our results are confirmed by data from MCF-7/UGCG Kd cells, which exhibit significantly lower total GlcCer, LacCer and ceramide levels, whereas total dh-ceramide levels are increased as compared to control cells (supplemental data 14). In addition, we determined the mRNA expression of the oncogene G*olgi phosphoprotein 3* (GOLPH3), which localizes in the Golgi apparatus and is involved in vesicle exit for trafficking to the plasma membrane. The mRNA level of GOLPH3 is decreased in MCF-7/UGCG OE cells as compared to control cells (Fig. 4C).

## Discussion

**Figure 5:**
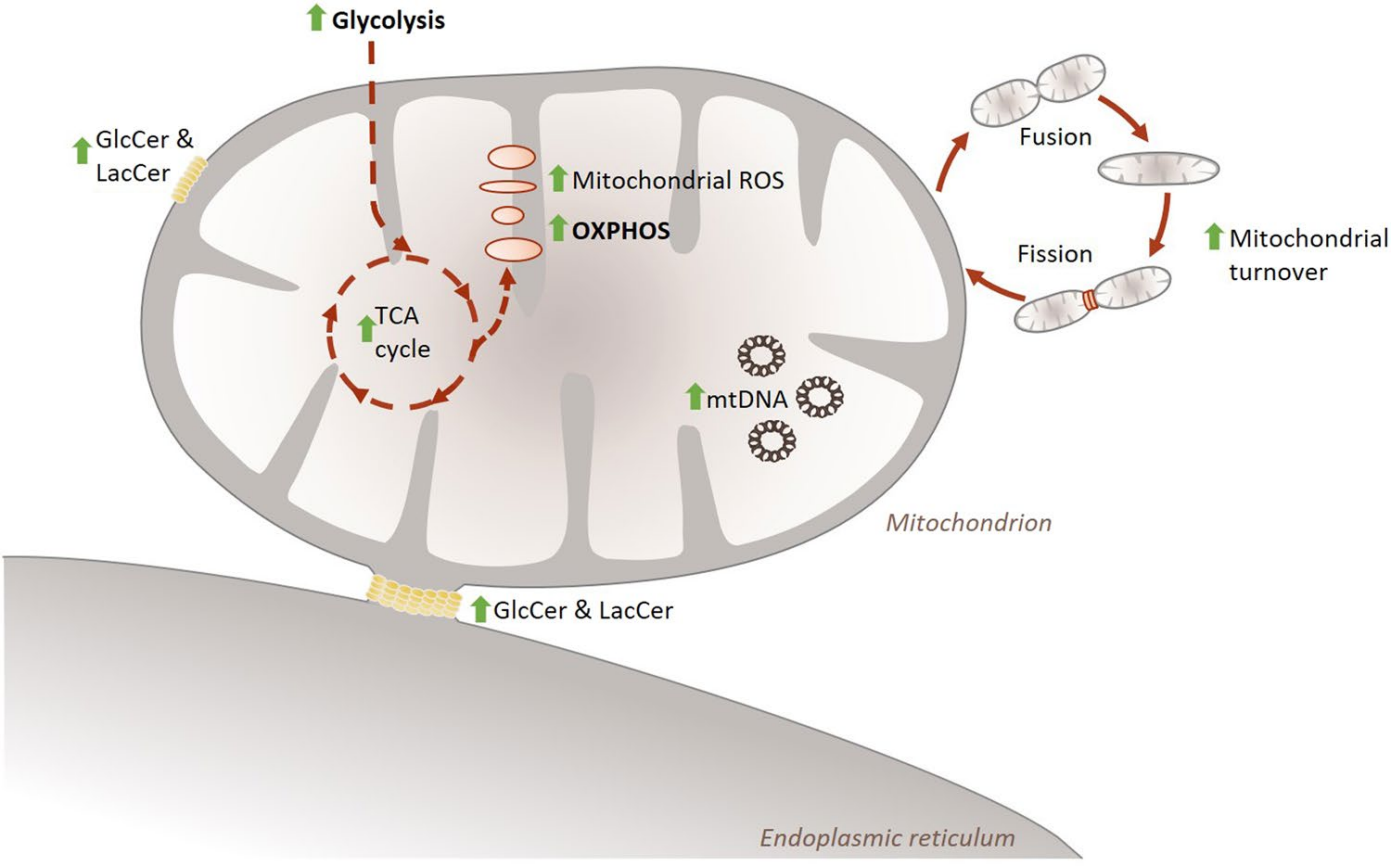
Schematic overview of the observed effects of UGCG overexpression on breast cancer cell metabolism. UGCG OE leads to increased glycolysis, fueled TCA cycle and increased OXPHOS. This leads to increased mitochondrial ROS level, which is accompanied by increased mitochondrial turnover. The effects are not ascribable to increased mitochondrial mass, but changes sphingolipid composition of ER/mitochondria fractions, which presumably contain ER-mitochondria contact sites. TCA = *tricarboxylic acid*, ROS = *reactive oxygen species*, OXPHOS = *oxidative phosphorylation*, GlcCer = *glucosylceramide*, LacCer = *lactosylceramide*, mtDNA = *mitochondrial DNA*, green arrow = increased in MCF-7/UGCG OE cells compared to control cells.

With this study we were able to show that UGCG *overexpression* (OE) mediates a metabolic transformation from a quiescent/aerobic to energetic breast cancer cell type indicated by increased glycolysis and OXPHOS. This is accompanied by changes in sphingolipid content in the ER/mitochondria fraction and increased mitochondrial turnover despite unchanged mitochondrial mass (Fig. 5).

UGCG OE mediates increased glycolysis in breast cancer cells. Glycolysis provides not only energy, but also macro molecules to generate biomass to maintain cell proliferation. Increased glycolytic flux does not automatically mean impairment of OXPHOS (reviewed in^26^). Indeed, we measured increased superoxide (presumably evolved by increased OXPHOS) and decreased mitochondrial membrane potential following UGCG OE, but we could clearly show that the oxidative metabolism is not impaired, but rather increased in UGCG overexpressing cells (Fig. 5). This shows that the increased glycolysis does not need to restore mitochondrial dysfunction and that this contributes to increased proliferation of MCF-7/UGCG OE cells^6^. Kao *et al*. also observed increased basal respiration and maximal respiratory capacity accompanied by increased UGCG expression in drug resistant HL-60 cells^27^. By using sphingolipid enzyme inhibitors the authors showed sphingolipid level alteration-mediated changes of mitochondrial function^27^. We confirmed our previous results, which showed increased flux through the TCA cycle following UGCG OE^22^ by increased OGDH (catalyzes a rate-limiting step in the TCA cycle) and GOT2 mRNA level and decreased GOT1 mRNA level. OGDH is a mitochondrial redox sensor, which can undergo S-glutathionylation following an increase in H_2_O_2_ levels^28^. In addition, the pyruvate dehydrogenase mRNA is more expressed following UGCG OE, which supports fuel the TCA cycle. However, elevated ^14^CO_2_ level from the uniformly labeled [U^14^C]-labelled glucose could also, at least partly, result from an increase in flux through the pentose phosphate pathway. We could already show an increase in NADPH concentration following UGCG OE in MCF-7 cells^22^.

Since OXPHOS and TOM20 protein concentration and NAO-positive cell number is unchanged following UGCG OE, we assume that the effects on glycolysis and oxidative metabolism are not ascribable to increased mitochondrial mass. Interestingly, UGCG Kd in MCF-7 cells leads to reduced ATP synthase protein concentration indicating that the effect leading to increased ATP synthesis in MCF-7/UGCG OE cells is UGCG-dependent. However, MCF-7/UGCG OE cells exhibit a significantly higher mtDNA copy number than MCF-7/empty control cells. mtDNA exhibits high mutation rates compared to genomic DNA potentially due to persistent exposure to mutagenic ROS resulting from respiration (reviewed in^29^). Since MCF-7/UGCG OE cells exhibit increased mitochondrial ROS, it is likely that this is the cause for increased mtDNA amount. Hori *et al*. showed that redistribution of the electron transport chain during the fusion process leads to increased ROS level, which resulted in increased mtDNA copy number^30^. Increased mtDNA content provides sufficient mtDNA copies for persistently running of fusion and fission processes and prevents production of mitochondria without mtDNA (reviewed in^31^). The phenomenon of altered mtDNA content is present in various human malignancies (reviewed in^32^). mtDNA copy increase was shown in various cancer types such as colorectal carcinoma, prostate and ovarian cancer, while in for example hepatocellular carcinoma the mtDNA copy number is decreased (reviewed in^32^). In contrast to our data, Fan *et al*. showed a decrease in mtDNA copy number in breast cancer patients^33^. On the other hand, an increased mtDNA content was revealed in drug resistant human laryngeal cancer cells^34^.

We checked the expression of several enzymes involved in the process of mitochondrial fusion and fission to investigate whether or not one of the processes is favored following UGCG OE. Surprisingly, UGCG OE does not lead to increased fusion or fission but might be leading to increased mitochondrial turnover. This is shown by increased respectively decreased counteracting enzyme mRNA or protein expression and data are confirmed by results, which show no significant alterations on mRNA level of proteins involved in mitochondrial fusion and fission processes following UGCG Kd in MCF-7 cells as compared to control cells. PINK1 assesses the internal state of mitochondria by being quickly disposed when the inner membrane potential of the mitochondria is high. Unhealthy mitochondria fail to import and to degrade PINK1 leading to accumulation of the kinase on the cytosolic site, which represents mitochondrial dysfunction. Cytosolic site located PINK1 recruits Parkin leading to ubiquitination of outer mitochondrial membrane proteins (reviewed in^35^). Since PINK1 mRNA expression is increased following UGCG OE, it is possible that PINK1 synthesis is increased due to the decreased mitochondrial membrane potential we detected. Parkin mRNA expression is unaltered, but the protein could still be recruited by PINK1 to tag these mitochondria leading to increased fission rate. If this would be the case, we would have expected low MFN1 and OPA1 (pro-fusion proteins) production, because both proteins are blocked by the PINK1/Parkin signaling pathway. But we measured increased OPA1 protein concentration, whereas MFN2 is reduced on protein level. Pallanck discusses the model of PINK1 accumulation in damaged mitochondria leading to MFN2 phosphorylation, subsequent Parkin recruitment and mitochondrial degradation (reviewed in^36^). MFN2 is also considered anti-tumorigenic and expressed at low levels in various human cancers, including MCF-7 breast cancer cell line^37^. Following UGCG OE, MFN2 protein levels are even more decreased, which would indicate less mitochondrial damage and degradation respectively. This is also shown by increased oxidative metabolism in MCF-7/UGCG OE cells. That MFN1 and MFN2 exhibit functional differences is also shown by higher GTPase activity of MFN1 as compared to MFN2 and higher affinity of MFN2 for GTP as compared to MFN1^38^. Increases of MFN1 and OPA1 were related to an increase in OCR and ATP levels in human normal dermal fibroblasts during aging^39^. Furthermore, FIS1 (pro-fission protein) mRNA expression is increased, whereas total DRP1 and phospho-DRP1 (Ser616) protein levels are unchanged. Phosphorylation of DRP1 at Ser616 usually promotes mitochondrial fission^40^. In summary, we cannot state whether or not the process of fusion or fission in mitochondria is increasingly executed. Presumably, increased superoxide levels following UGCG OE lead to reduced mitochondrial membrane potential and results in increased mitochondrial turnover (Fig. 5). Increased respiration, superoxide level and fusion-fission rate (increased OPA1 and DRP1 production) are also shown in K562 (chronic myelogenous leukemia) cells by Ruiz *et al*. The authors assume that increased mitochondrial turnover can lead to smaller, condensed and “boosted” mitochondria resulting in increased cell proliferation^41^.

We confirmed our previous results from the study in 2018^6^, which showed an accumulation of the ER marker PDI in fraction 3, 5, 6, 7 and 9 (sucrose density centrifugation). These fractions also exhibit increased GlcCer level as compared to control cells^6^. Hence, together with the results of this study, we could show that UGCG OE leads to accumulation of GlcCer in specific regions of the plasma membrane and to GlcCer and LacCer accumulation in the ER/mitochondria fraction (Fig. 5) The observed UGCG mediated effects on mitochondrial enzyme activity (OXPHOS) might be triggered by changed lipid composition of the ER/mitochondria fraction, which contains ER-mitochondria contact sites. Intriguingly, UGCG Kd in MCF-7 cells abolishes the effect of UGCG OE on lipid composition in ER/mitochondria fractions and leads to reduced ATP synthase protein concentration in mitochondria. We assume that this finding leads to the effect of reduced cell proliferation of MCF-7/UGCG Kd cells, we showed in our study in 2018^6^. In 2010, Ciarlo *et al*. showed that upon UGCG inhibition with [D]-PDMP, the recruitment of fission molecules to the mitochondria was impaired and consequently fission was reduced in lymphoid cells. Furthermore, apoptosis was decreased^42^. These findings likewise indicate an influence of the UGCG on mitochondrial raft-like structures, but also indicate a cell-type specific influence of UGCG on cellular energy metabolism. Since we already showed UGCG OE-dependent alterations of the GEM composition of the plasma membrane of MCF-7 cells^6^, it is likely that alterations of intracellular membranes are occurring as well. The functionally and structurally very different inner and outer mitochondrial membranes highly influence the function of the organelles (reviewed in^43^). Furthermore, it was shown that ER-mitochondria contact sites, which are important for mitochondrial dynamics and morphology, are influenced by lipid raft-like structures (reviewed in^5^). In addition, these contact sites coordinate the licensing of mtDNA replication, which is increased in MCF-7/UGCG OE cells with division to distribute newly replicated nucleosides to daughter mitochondria^44^. Even though the sphingolipid concentration in the mitochondrial membranes is low, its influence is huge. Accumulation of ceramide leads to the formation of large channels in the mitochondrial outer membrane^45^ leading to pro-apoptotic protein release during the induction phase of apoptosis. It is known that mitochondria exhibit an independent ceramide pool, whereas it is not clear from where they are delivered (reviewed in^46^). However, the finding that the total ceramide level in the ER/mitochondria fractions of the more viable MCF-7/UGCG OE cells is not changed as compared to control cells, indicates that mitophagy is not induced (autophagy induction could be excluded^22^). Interestingly, specifically C_18:0_-Cer concentration is increased in ER/mitochondria fractions of MCF-7/UGCG OE cells. We already showed that the C_18:0_-Cer synthetizing *ceramide synthase 1* (CerS1), whose trafficking from ER to mitochondria mediates cellular stress and cell death^47^, is not expressed in MCF-7 cells^48^. CerS4 is also able to produce C_18:0_-Cer, but its mRNA expression is reduced following UGCG OE^9^. Accordingly, the increased C_18:0_-Cer concentration could be related to increased *dihydroceramide desaturase* (DES) activity. Contradictory, we also measured increased C_18:0_-dh-Cer level. C_18:0_-dh-Cer is a substrate for DES and we assumed that the concentration is would be decreased, which is contradictory. To our knowledge, the gangliosides GM1 and GD3 are the only complex GSL, which are related to mitochondrial lipid raft-like structures so far (reviewed in^5,49^). The interaction of GD3 with mitochondrial lipid raft-like structures leads to changes in the mitochondrial membrane potential presumably via enhancing ROS formation of complex III of the mitochondrial electron transport chain and subsequent opening of mitochondrial permeability transition pore and cytochrome c-dependent activation of caspase 3 (reviewed in^5^). Since GM1 and GD3 are downstream of GlcCer, complex GSL might also be responsible for the observed changes following UGCG OE. However, it is unclear how GSL synthesized in the Golgi apparatus reach the ER and/or mitochondria. Annunziata *et al*. propose redistribution of GM1 in the ER membrane by contact between ER membranes and the plasma membrane (reviewed in^49^). Noteworthy, *b*-series derived gangliosides such as GD3 and GD2 exhibit tumor-enhancing function and *a*-series derived gangliosides such as GM1, GM2 and GM3 often exhibit tumor-suppressing function (reviewed in^2^).

GOLPH3 is a membrane protein localized in the *trans*-Golgi and in vesicles budding from the *trans*-Golgi. Amongst others, GOLPH3 specifically binds phosphatidylinositol-4-phosphate, which promotes vesicle exit for trafficking to the plasma membrane^50^. The oncogene GOLPH3 was found to be involved in the progression of various solid tumors, amongst others breast tumors^51^, and related to poor clinical outcome in many cancers. In contrast to these findings, the GOLPH3 gene expression is reduced in MCF-7/UGCG OE cells. A GOLPH3 knockdown causes scission of the interconnecting Golgi ribbon tubules which leads to the division of the individual Golgi cisternae stacks. Consequently, the vesicular transport deriving from these stacks is decreased (reviewed in^52^). A GOLPH3 decrease-mediated reduction of the vesicular transport in MCF-7/UGCG OE cells might be an explanation for the GEM restricted (not in the whole plasma membrane) GSL level increase which was described by us in 2018^6^. Wang *et al*. revealed that GOLPH3 modulates the sensitivity towards the chemotherapeutic *5-fluoruracil* (5-FU). More precisely, an increased GOLPH3 expression correlates with an advantageous prognosis in patients treated with 5-FU and predicts a higher sensitivity towards 5-FU in colorectal cancer cells^53^. Since the GOLPH3 expression is reduced in MCF-7/UGCG OE cells, which are less sensitive towards chemotherapeutic treatment^6^, the findings of Wang *et al*. are in line with our results.

Our data indicate that UGCG is closely connected to the breast cancer cell energy metabolism. This connection allows the cells to execute increased glycolysis and OXPHOS, which maintains sufficient supply with energy and building blocks needed for augmented cell proliferation. Molecular trigger might be changes in the sphingolipid composition of ER/mitochondria fraction leading to increased OXPHOS protein activity. This is accompanied by accelerated mitochondrial turnover (Fig. 5).

Since we could show that UGCG leads to glutamine dependency in MCF-7 cells, which results in increased glycolysis, OXPHOS (current study) and cell proliferation^22^, pharmacological inhibition of both glutamine metabolism and GlcCer synthesis via UGCG could be beneficial for treatment of breast cancer patients. This would be a novel therapeutic strategy for breast cancer treatment. Several inhibitors of glutamine metabolism such as *benzylserine and L-γ-glutamyl-p-nitroanilide* (GPNA, glutamine transporter inhibitor) or CB-839 (glutaminase I inhibitor) are available but are either low in efficacy or toxic (reviewed in^54^). Potential side effects of GPNA, CB-839 or others could be reduced by lowering the dose and drug efficacy could be increased by co-treatment with UGCG inhibitors, because we assume that both inhibitors would work synergistically in the process of inhibiting breast cancer cell proliferation.

## Supplementary information

Supplementary Information.
