# Supplementary material for: UGCG overexpression leads to increased glycolysis and increased oxidative phosphorylation of breast cancer cells
Source: Sci Rep. 2020 May 18;10:8182. doi: 10.1038/s41598-020-65182-y (PMC7234995; doi:10.1038/s41598-020-65182-y)
Supplement: Supplementary file 1 — Supplementary Information. [file 41598_2020_65182_MOESM1_ESM.pdf]

UGCG overexpression leads to increased glycolysis and increased oxidative phosphorylation of breast cancer cells

Nina Schömel[1], Lisa Gruber[1], Stephanie J. Alexopoulos[2], Sandra Trautmann[1], Ellen M. Olzomer[2], Frances L. Byrne[2], Kyle L. Hoehn[2], Robert Gurke[1,3], Dominique Thomas[1], Nerea Ferreirós[1], Gerd Geisslinger[1,3], Marthe-Susanna Wegner[1,2]

[1]*pharmazentrum frankfurt*/ZAFES, Institute of Clinical Pharmacology, Johann Wolfgang Goethe University, Theodor Stern-Kai 7, 60590 Frankfurt am Main, Germany.

[2]School of Biotechnology and Biomolecular Sciences, University of New South Wales, Sydney, New South Wales 2052, Australia.

[3]Fraunhofer Institute for Molecular Biology and Applied Ecology IME, Project Group Translational Medicine and Pharmacology (TMP), Theodor Stern-Kai 7, 60590 Frankfurt am Main, Germany.

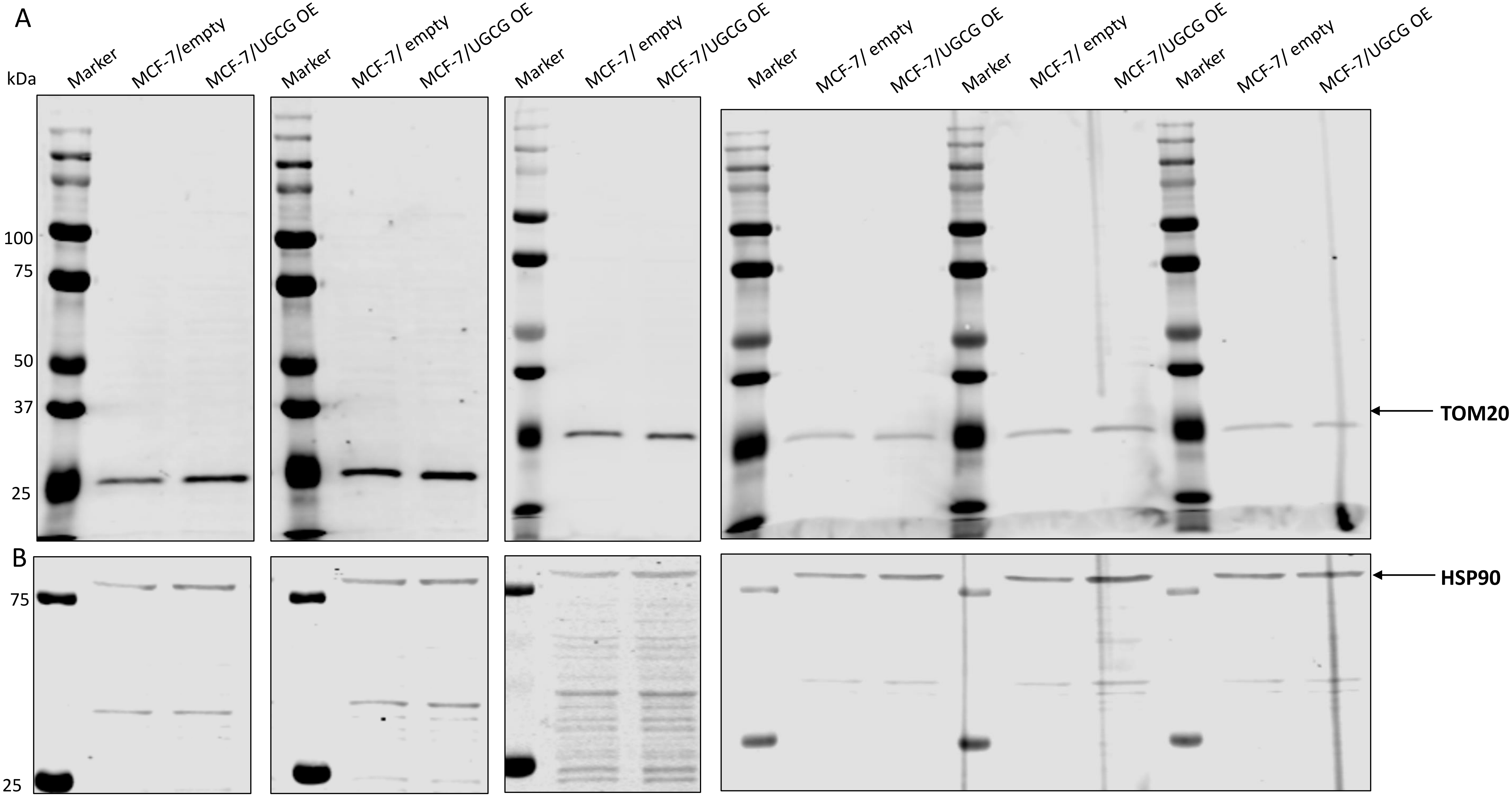

Analysis mitochondrial dynamics by Western Blot. (**A**) *Translocase of the outer mitochondrial membrane 20* (TOM20) protein Western Blots of all experiments. (**B**) *Heat shock protein 90* (HSP90) protein Western Blots of all experiments.

Supplemental data 1

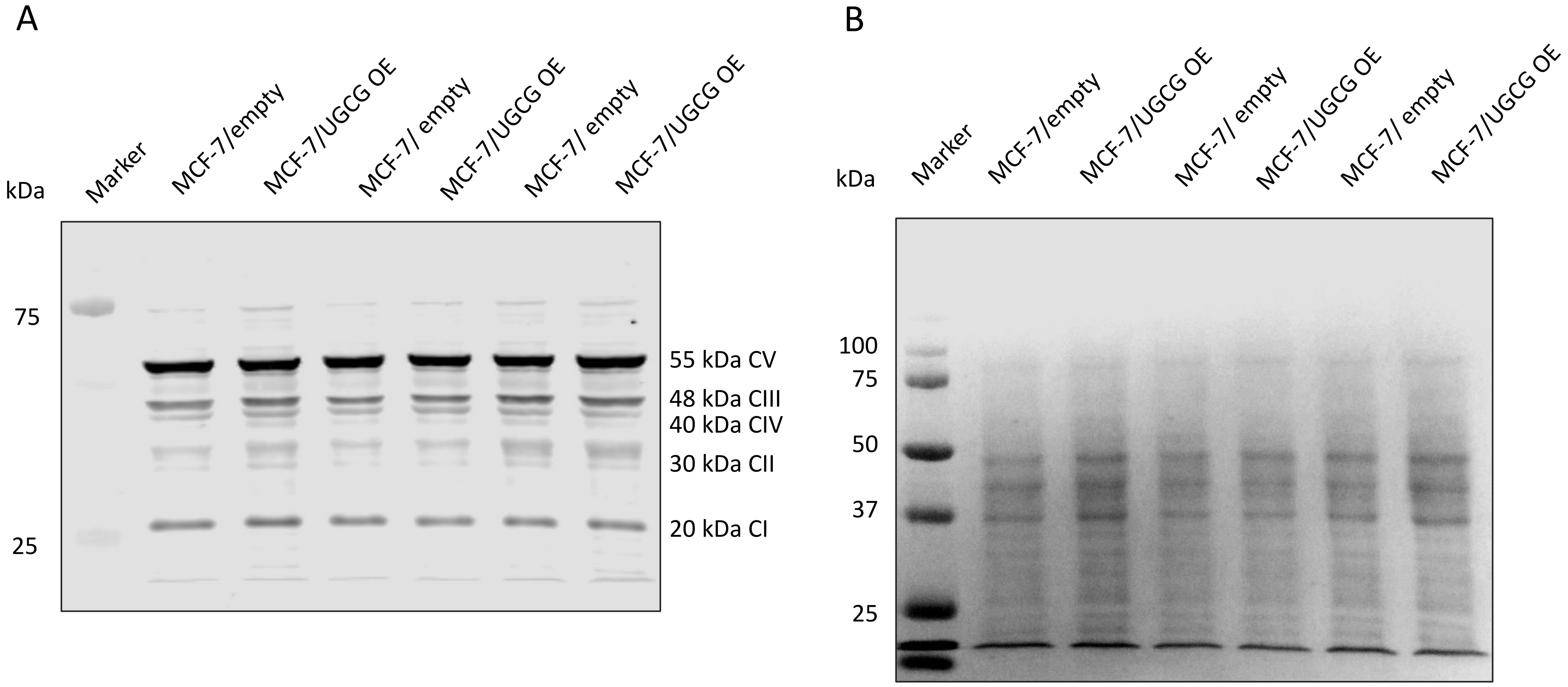

Analysis of OXPHOS protein concentrations by Western Blot. (**A**) OXPHOS protein Western Blots of all experiments. (**B**) Ponceau dye of all experiments. C = complex.

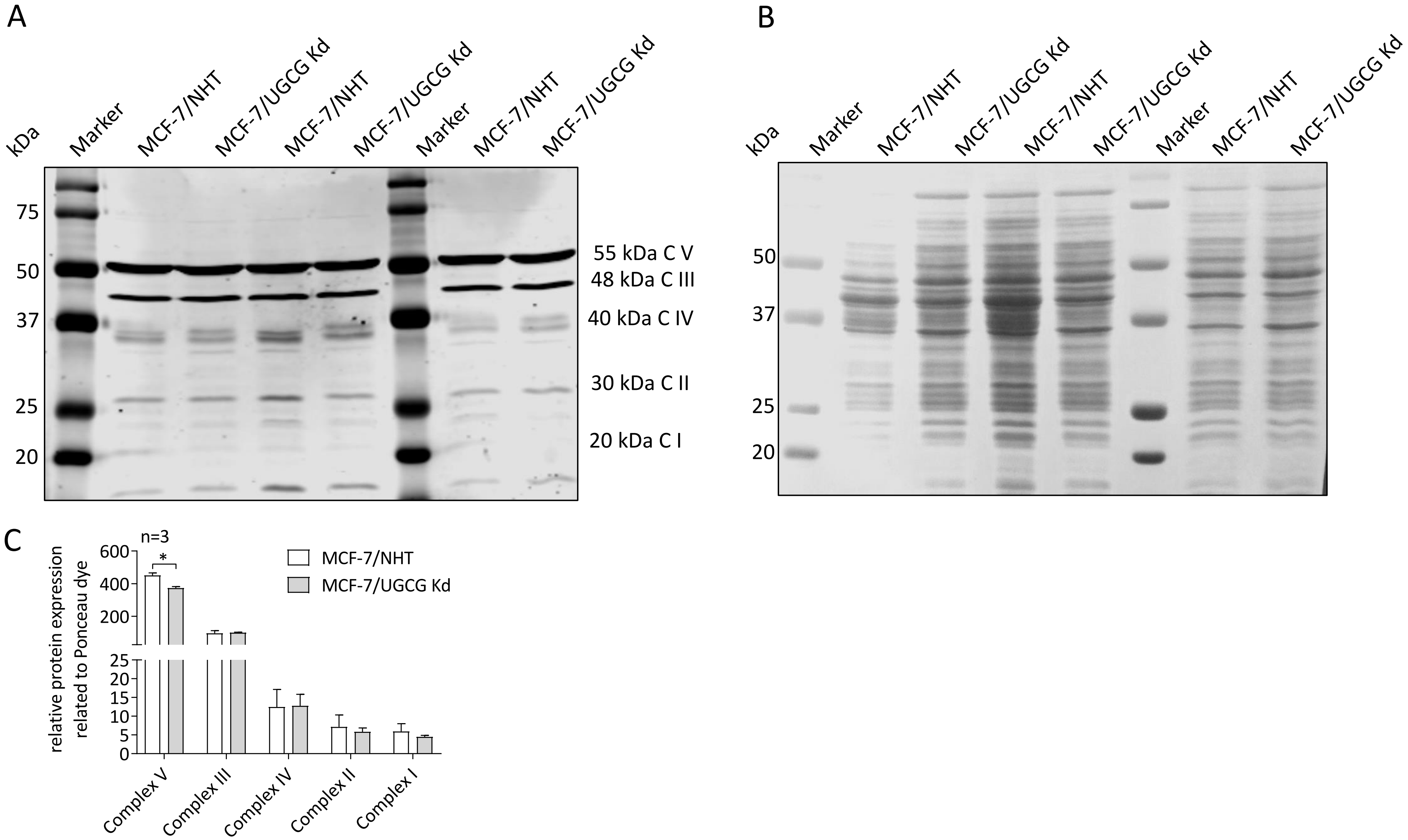

Determination of OXPHOS protein concentrations by Western Blot analysis. MCF-7/UGCG knockdown (Kd) cells were generated as described previously [6]. NHT = non-human target control cells. C = complex. (**A**) OXPHOS protein Western Blots of all experiments. (**B**) Ponceau dye of all experiments. (**C**) Densitometrical analysis of OXPHOS complexes I-V protein concentrations by Western blot analysis. Protein expression is related to Ponceau dye. Data are presented as a mean of n = 3 ± SEM. , ***p ≤ 0.001..

Supplemental data 3

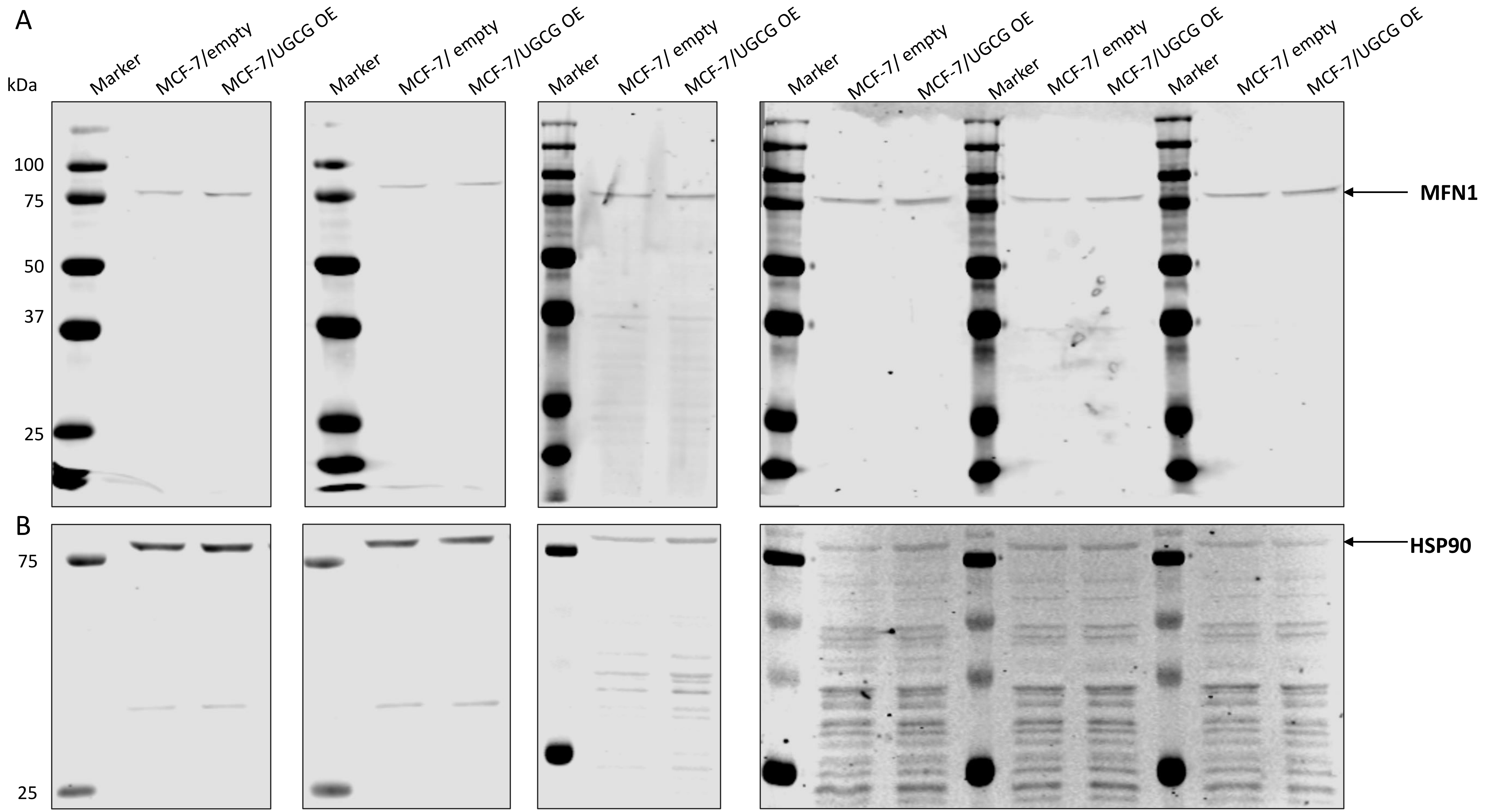

Analysis mitochondrial dynamics by Western Blot. (**A**) *Mitofusin 1* (MFN1) protein Western Blots of all experiments. (**B**) *Heat shock protein 90* (HSP90) protein Western Blots of all experiments.

Supplemental data 4

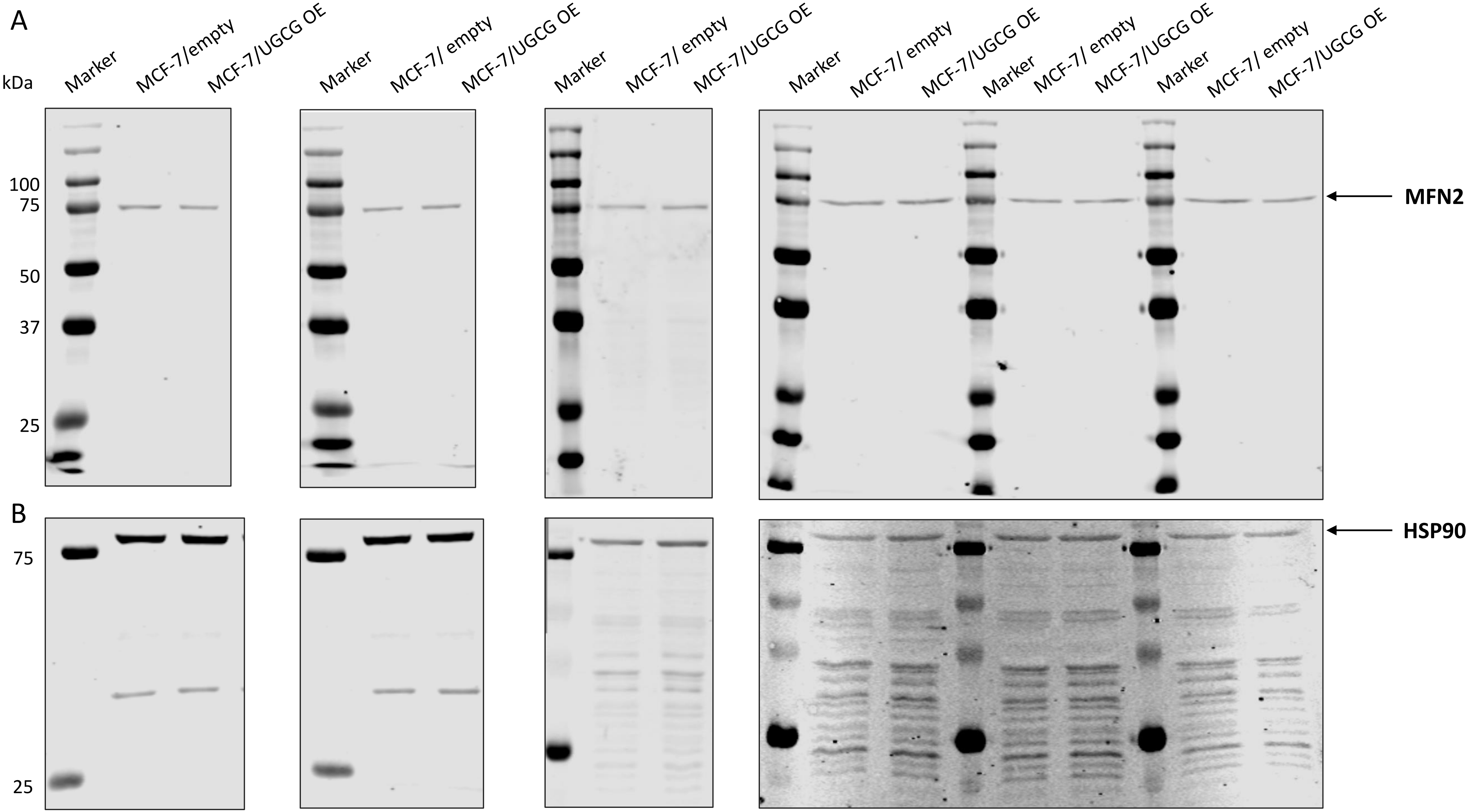

Analysis mitochondrial dynamics by Western Blot. (**A**) *Mitofusin 2* (MFN2) protein Western Blots of all experiments. (**B**) *Heat shock protein 90* (HSP90) protein Western Blots of all experiments.

Supplemental data 5

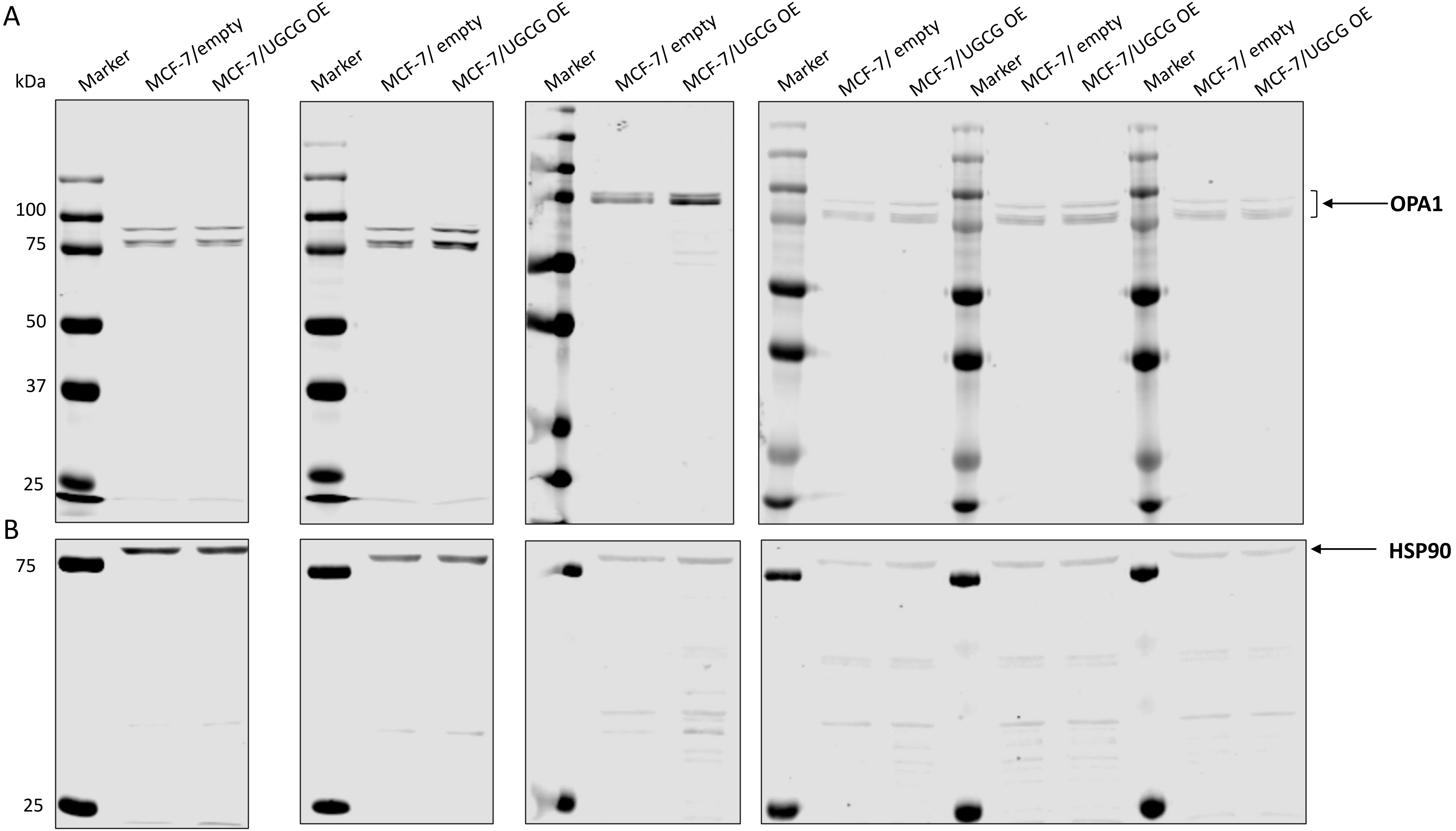

Analysis mitochondrial dynamics by Western Blot. (**A**) *Optic atrophy type 1* (OPA1) protein Western Blots of all experiments. (**B**) *Heat shock protein 90* (HSP90) protein Western Blots of all experiments.

Supplemental data 6

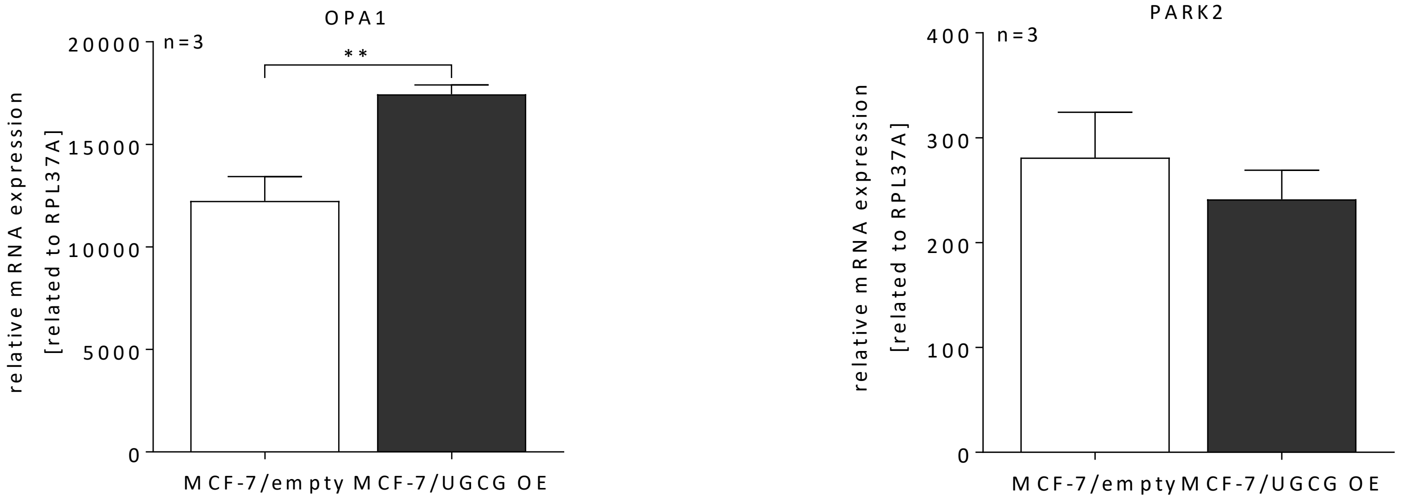

Analysis of *optic atrophy type 1* (OPA1) and *Parkin 2* (PARK2) mRNA expression by qRT-PCR. mRNA expression is related to the housekeeper RPL37A. Data are presented as a mean of n = 3 ± SEM. **p ≤ 0.01.

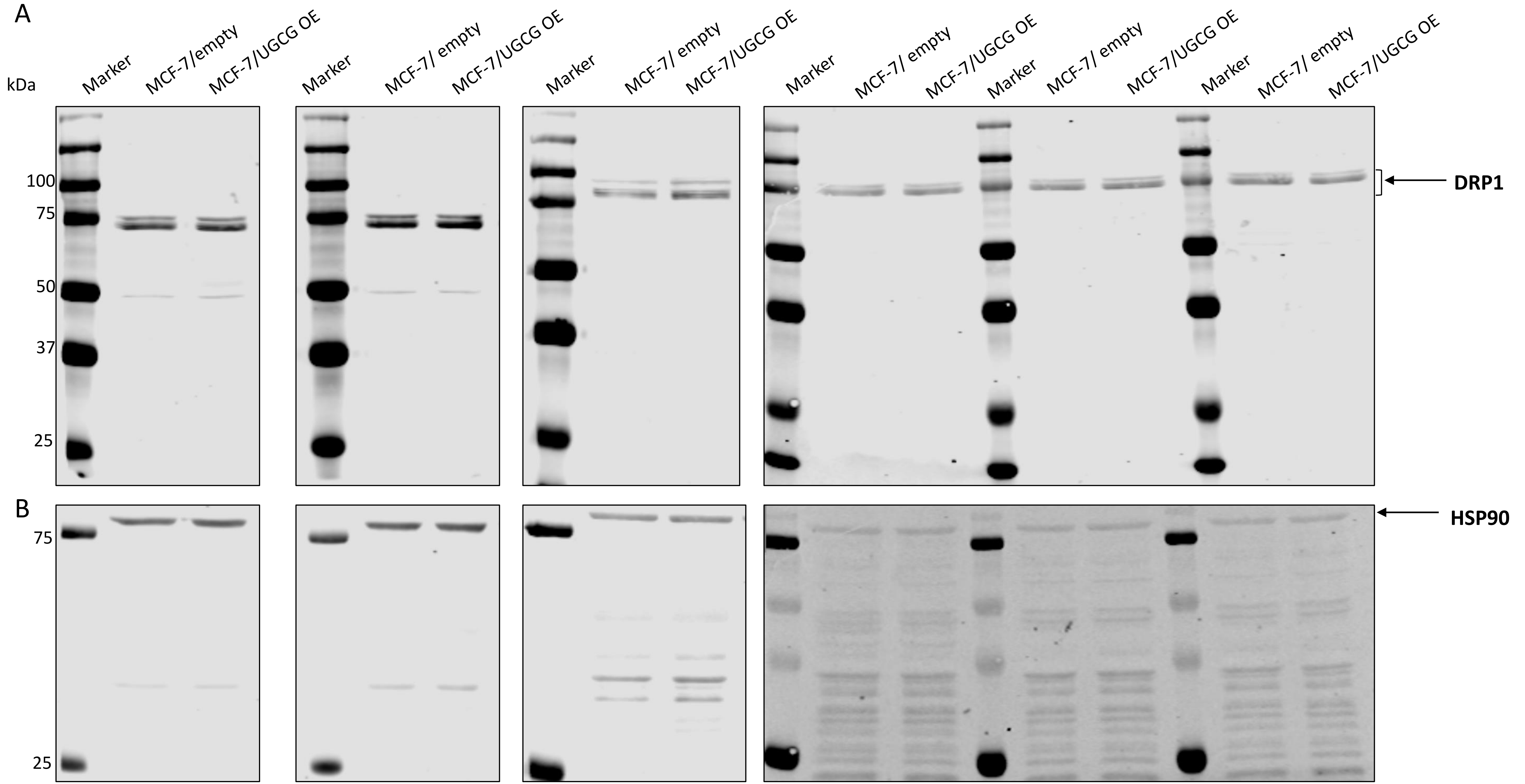

Analysis mitochondrial dynamics by Western Blot. (**A**) *Dynamin related protein1* (DRP1) protein Western Blots of all experiments. (**B**) *Heat shock protein 90* (HSP90) protein Western Blots of all experiments.

Supplemental data 8

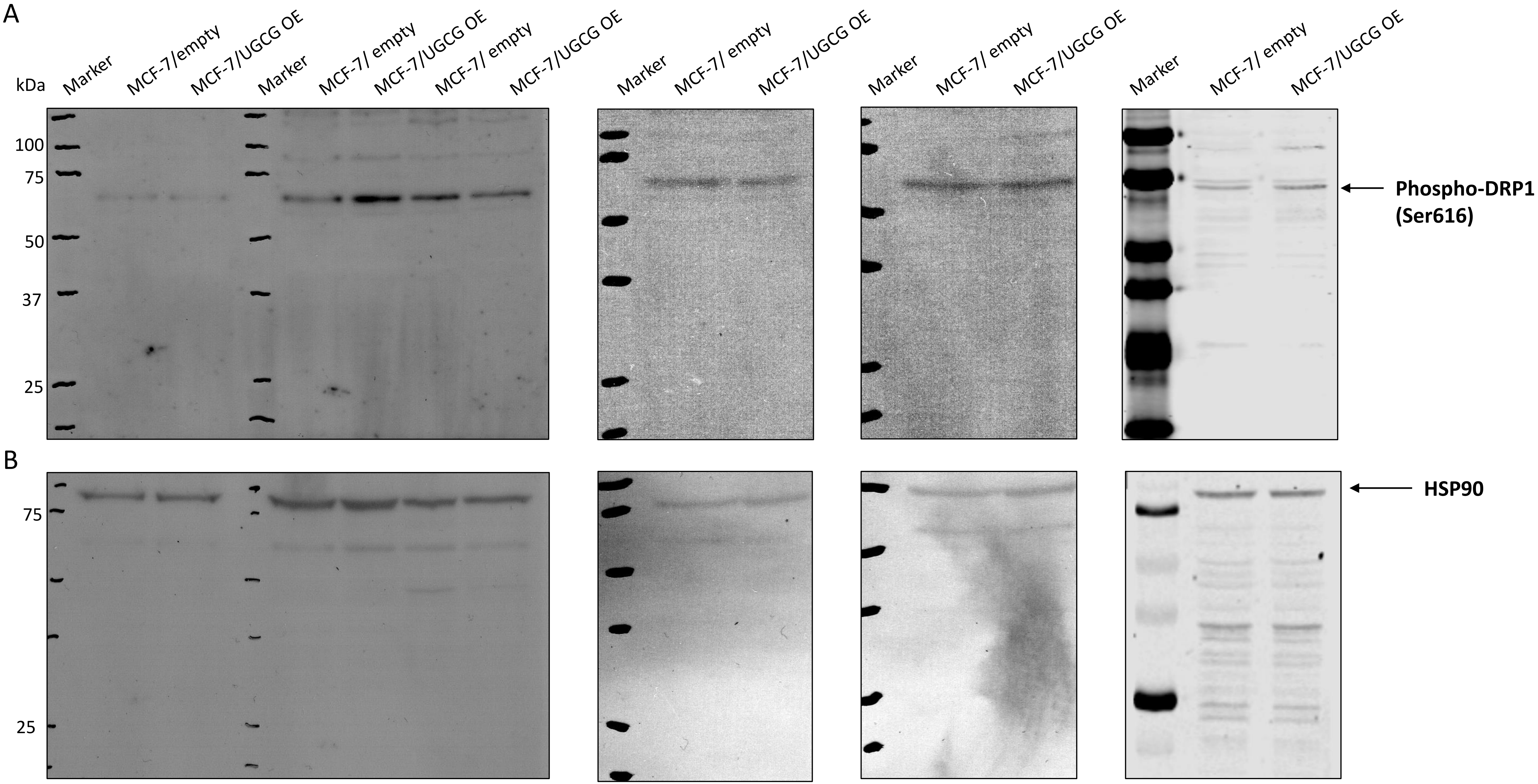

Analysis mitochondrial dynamics by Western Blot. (**A**) *Phospho-dynamin related protein1* (Phospho-DRP1) (Ser616) protein Western Blots of all experiments. (**B**) *Heat shock protein 90* (HSP90) protein Western Blots of all experiments.

Supplemental data 9

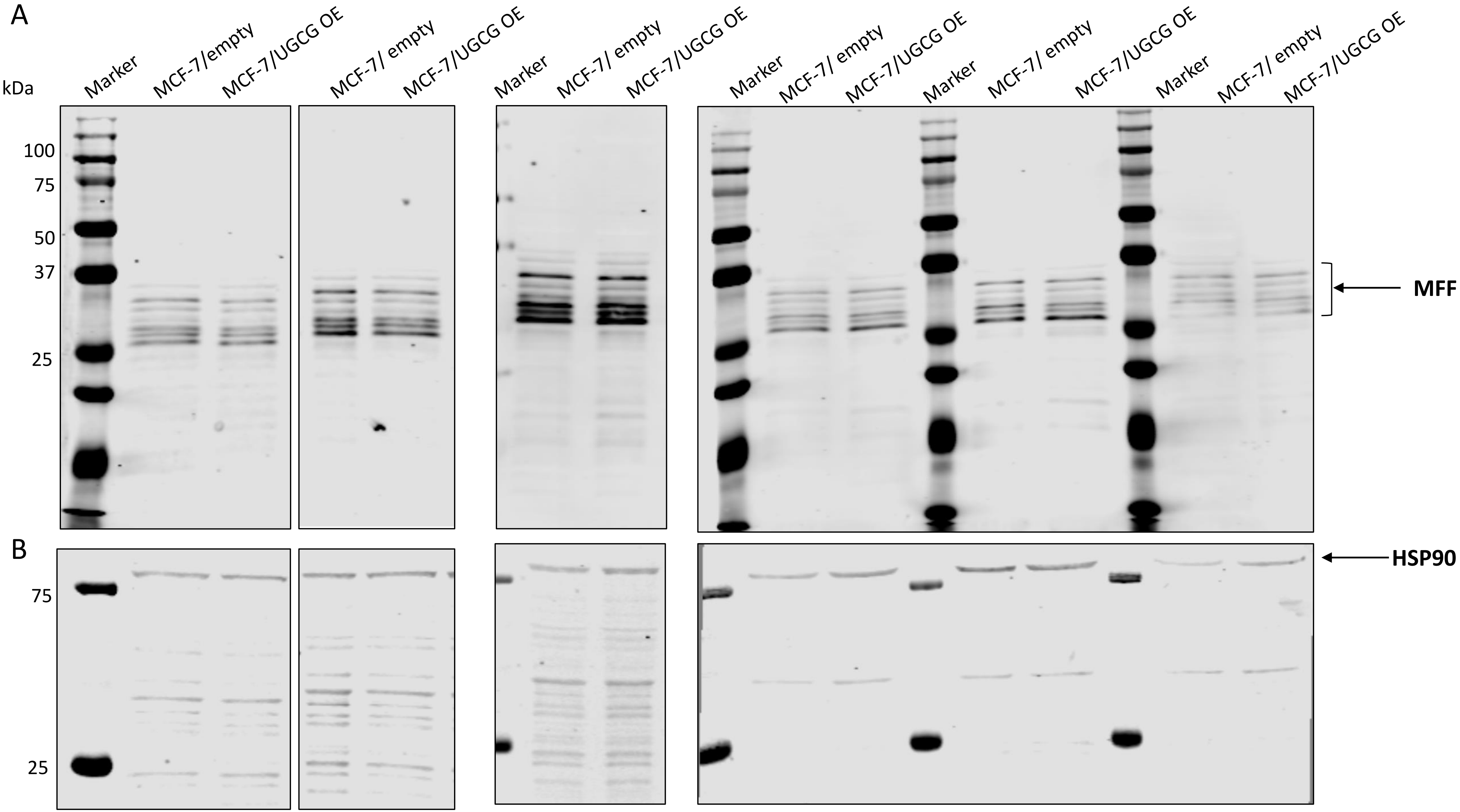

Analysis mitochondrial dynamics by Western Blot. (**A**) *Mitochondrial fission factor* (MFF) protein Western Blots of all experiments. (**B**) *Heat shock protein 90* (HSP90) protein Western Blots of all experiments.

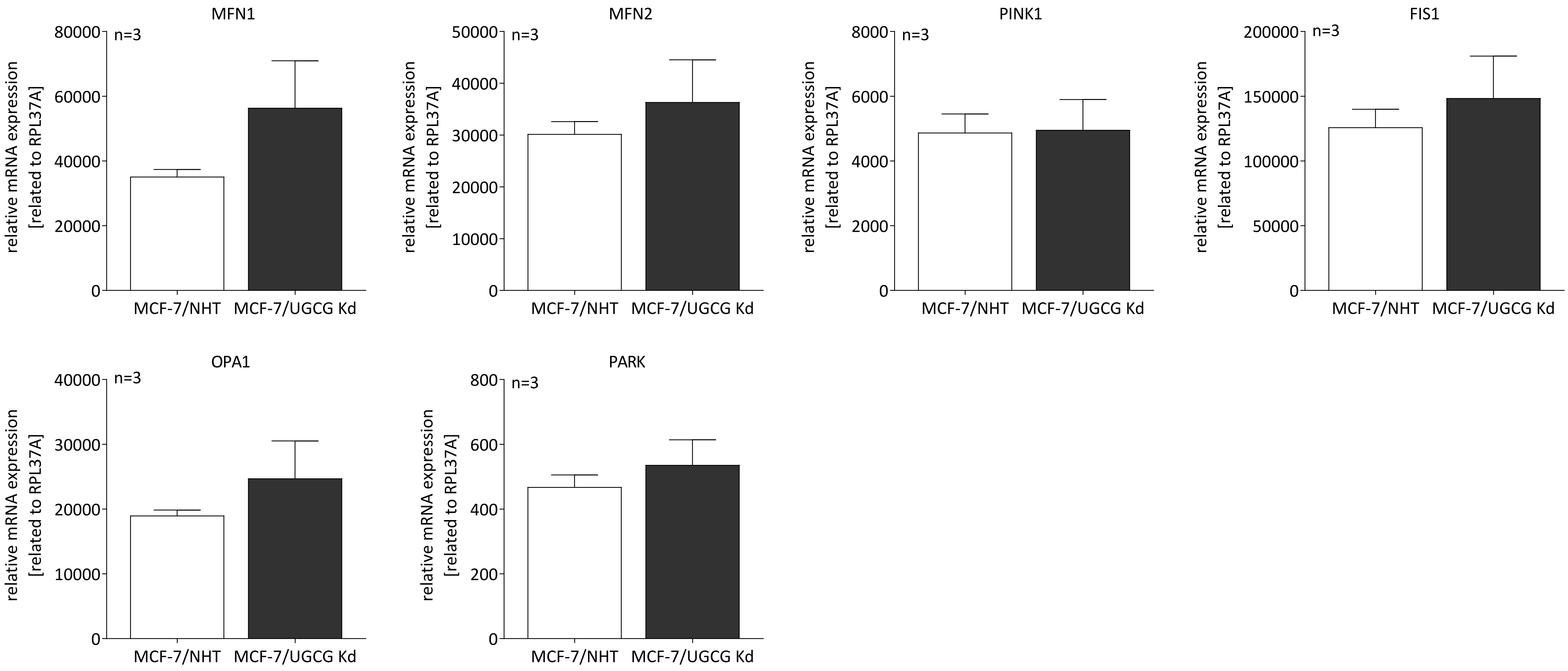

Analysis of mRNA expression of fusion and fission proteins in MCF-7/UGCG Kd cells by qRT-PCR. NHT = non-human target control, MFN1 = mitofusin 1, MFN2 = mitofusin 2, PINK1 = PTEN-induced kinase 1, FIS1 = mitochondrial fission protein 1, OPA1 = optic atrophy 1, PARK = parkin 2. mRNA expression is related to the housekeeper RPL37A. Data are presented as a mean of n = 3 ± SEM.

Supplemental data 11

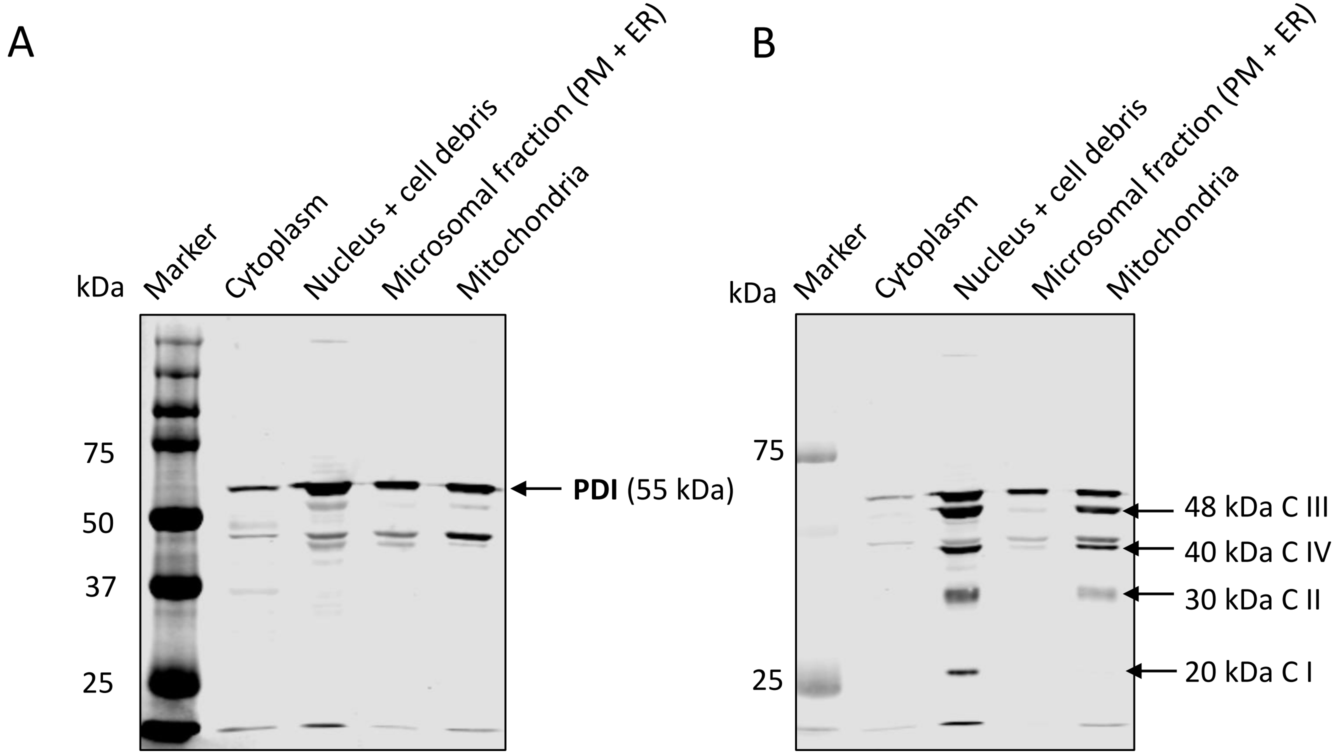

ER/mitochondria fraction verified by PDI and OXPHOS protein detection. (**A**) Representative image of anti-*protein disulfide isomerase* (PDI; ER marker) and (**B**) anti-Oxphos Rodent Western Blot Cocktail (mitochondria marker). PM = plasma membrane, ER = endoplasmic reticulum, C = Complex.

Material and Methods: Western blot analysis of purity of ER/mitochondria fractions. Isolated fractions were separated by 12 % SDS-PAGE as described in section *Protein concentration determination by Western blot analysis*. Anti-PDI antibody (ab2792, Abcam, Cambridge, UK) (1:250 in blocking solution was applied over night at 4 °C). The IRDye®680 conjugated secondary antibody (LI-COR Biosciences, Bad Homburg, Germany), and anti-OXPHOS rodent Western Blot Cocktail were applied as described in section *Protein concentration determination by Western blot analysis*.

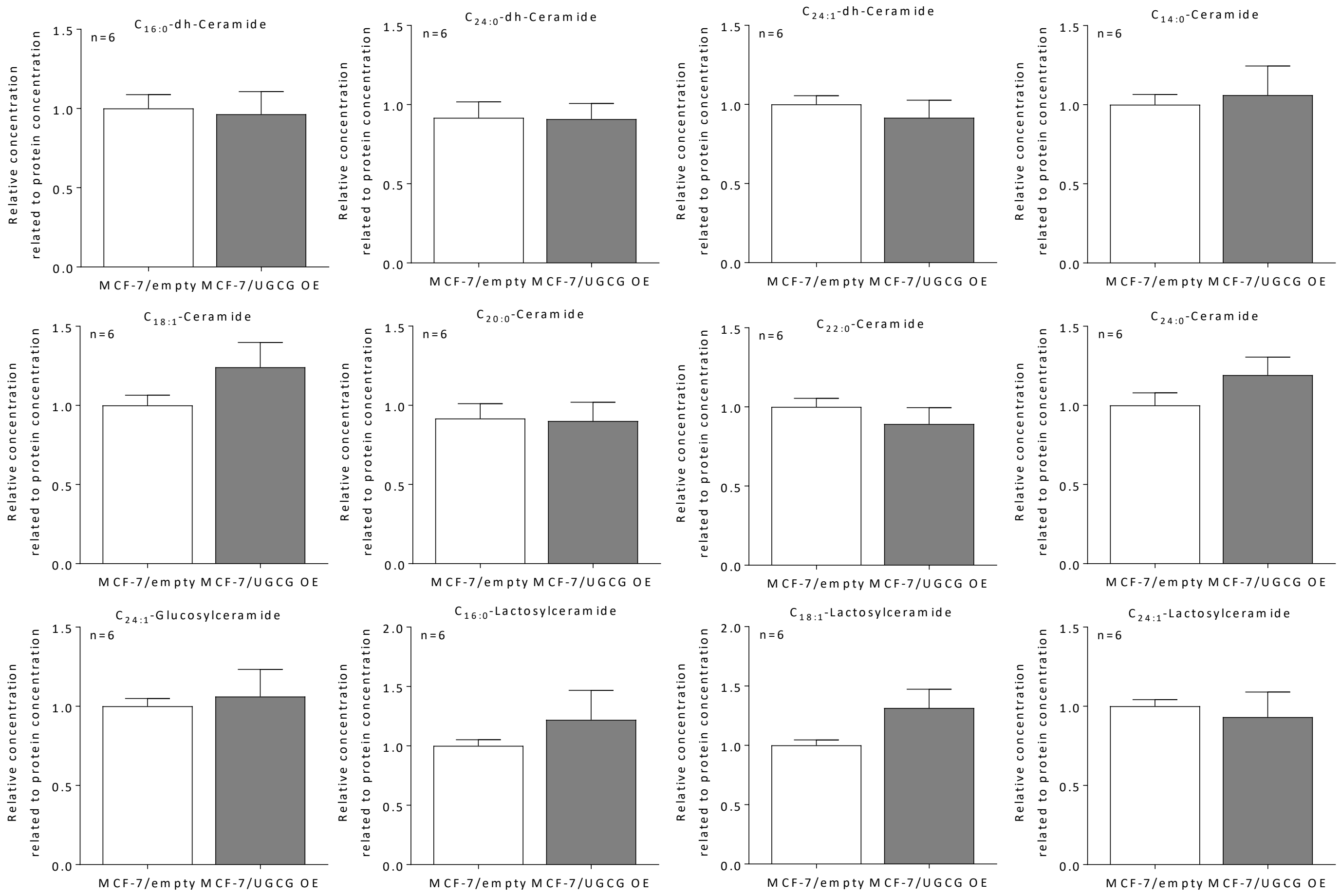

Sphingolipid levels of mitochondria/ER fractions, which were not changed following UGCG overexpression. Levels were related to MCF-7/empty. dh-Ceramide = dihydro-ceramide. Data are presented as a mean of n = 6 ± SEM.

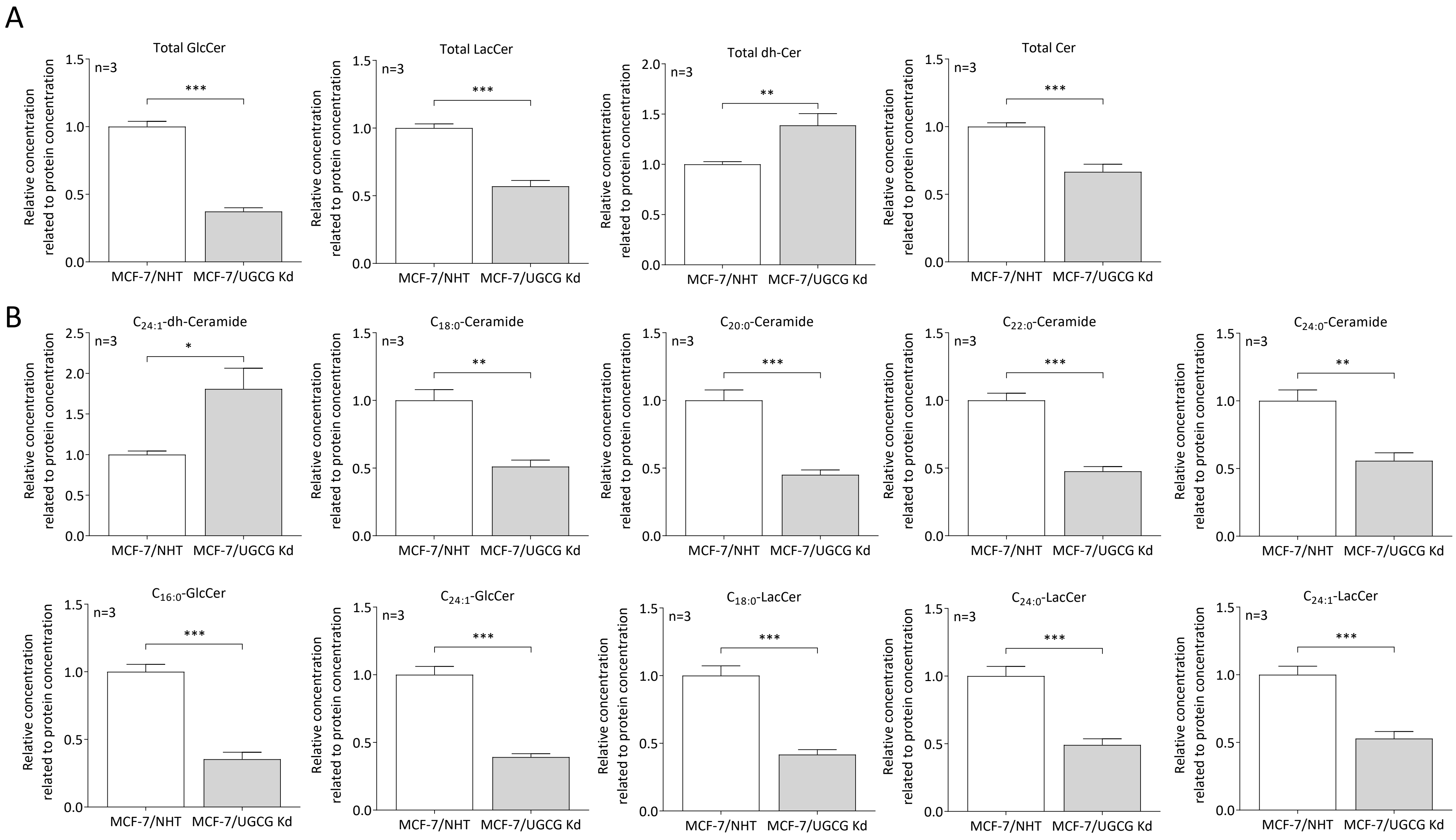

Sphingolipid levels of mitochondria/ER fractions following UGCG Kd. NHT = non-human target control. Levels were related to MCF-7/NHT. (**A**) Total glucosylceramide (GlcCer), lactosylceramide (LacCer), duhydro-ceramide (dh-Cer) and ceramide levels. (**B**) Analytes, which were significantly altered in MCF-7/UGCG Kd cells as compared to control cells. Data are presented as a mean of n = 3 ± SEM. *$p \leq 0.05$, **$p \leq 0.01$, ***$p \leq 0.001$.
